# Harnessing the gut microbiome to combat tuberculosis: a technological and clinical review

**DOI:** 10.3389/fcimb.2026.1847443

**Published:** 2026-06-19

**Authors:** Weiguo Sun, Meng Xiao, Syed Luqman Ali, Chanyuan Jin, Asifullah Khan, Shakirullah, Ruizi Ni, Yajing An, Mingming Zhang, Yuan Tian, Shradha Kaushik, Yuhang Zhang, Wenping Gong

**Affiliations:** 1 Beijing Key Laboratory of Emergency Vaccine Development and Process Translation, Senior Department of Tuberculosis, Chinese PLA General Hospital, Beijing, China; 2 Computer Network Information Center, Chinese Academy of Sciences, Beijing, China; 3 DUKE-NUS Medical School, National University of Singapore, Singapore, Singapore; 4 Department of Biochemistry, Abdul Wali Khan University Mardan, Mardan, KPK, Pakistan; 5 2nd Dental Center, Peking University School and Hospital of Stomatology, Beijing, China; 6 Department of Zoology, Abdul Wali Khan University Mardan, Mardan, KPK, Pakistan; 7 Graduate School, Hebei North University, Zhangjiakou, Hebei, China; 8 Department of Biomedical Sciences, University of Windsor, Windsor, ON, Canada; 9 Institute of Clinical Pharmacology, Peking University First Hospital, Beijing, China

**Keywords:** artificial intelligence (AI), gut microbiome (GM), metabolomics, next-generation sequencing (NGS), proteomics, tuberculosis (TB)

## Abstract

Tuberculosis (TB), especially multidrug-resistant and extensively drug-resistant strains, remains a severe global health threat. Advances in high-throughput sequencing, omics technologies and artificial intelligence have revealed the critical involvement of the gut microbiome (GM) in TB pathogenesis, diagnosis and treatment via the gut–lung axis. The GM modulates host immunity and metabolism; TB patients typically show reduced microbial diversity and enriched pro-inflammatory taxa closely linked to disease severity and treatment responses. Omics research has identified promising biomarkers and pathways for early diagnosis and personalized management, while artificial intelligence improves diagnostic accuracy and treatment outcome prediction. GM-targeted interventions, including probiotics, dietary adjustment and fecal microbiota transplantation, can enhance therapeutic efficacy and relieve adverse drug reactions. Current limitations include insufficient validation of the gut-lung axis’ causal mechanisms, lagged clinical translation of biomarkers, biases and errors in diagnosis and prediction, data privacy and security concerns, gaps in intervention research, and poor accessibility of related technologies in resource-scarce medical regions. Future studies need rigorous causal analyses, real-time monitoring tools and large-scale multicenter trials to validate microbiome-based strategies. This review highlights the translational potential of GM interventions to optimize personalized TB prevention, diagnosis and treatment and improve clinical outcomes.

## 1 Introduction

Tuberculosis (TB), caused by *Mycobacterium tuberculosis* (MTB) infection, is the leading cause of mortality from a single infectious disease worldwide (Gong and Du, 2025; Liu and Gong, 2025). According to the World Health Organization (WHO) 2025 Global Tuberculosis Report, more than ten million new TB cases are reported globally each year (WHO, 2025; Zhang et al., 2026). The continuous emergence of multidrug-resistant (MDR) and extensively drug-resistant (XDR) MTB strains poses formidable challenges to the traditional pathogen-centric diagnostic and therapeutic paradigm. On one hand, existing diagnostic tools exhibit insufficient sensitivity in key populations (Gong and Wu, 2021; Li et al., 2023), such as patients with sputum smear-negative TB, pediatric patients, and individuals living with human immunodeficiency virus (HIV) coinfection (Chaisson et al., 1996). On the other hand, the standardized six-month anti-TB treatment regimen shows substantial interindividual variability in efficacy (Grace et al., 2019), with adverse drug reactions and the emergence of drug resistance mutations further limiting treatment success rates. Against this backdrop, there is an urgent need to move beyond the conventional pathogen-centric model and adopt a systems medicine approach centered on host-microbiota-pathogen interactions.

Over the past decade, GM research has revealed the decisive role of the microbiota-immune-metabolic network in the pathogenesis of various chronic diseases, leading to the emergence of the “gut-lung axis” concept (Marsland et al., 2015; Enjeti et al., 2023; Harris and Marsland, 2025). The GM produces a repertoire of metabolites, including short-chain fatty acids (SCFAs), bile acids, and tryptophan derivatives, which remotely regulate the pulmonary immune microenvironment (Verma et al., 2024). Conversely, pulmonary MTB infection and anti-TB drug therapy can reshape GM composition, forming a bidirectional regulatory feedback loop (Chai et al., 2025; Liu et al., 2025). Recent multi-omics studies have consistently demonstrated that TB patients exhibit a characteristic GM dysbiosis—termed TB-associated dysbiosis—marked by reduced microbial diversity, depleted butyrate-producing bacteria, and enriched pro-inflammatory taxa. The severity of this microbial imbalance is strongly correlated with disease severity, treatment failure rates, and delayed immune reconstitution in TB patients. These findings indicate that the GM is not a passive bystander in TB pathogenesis but an active participant and a potential modulator of disease progression.

Technological breakthroughs have enabled the systematic dissection of GM-TB interactions. High-throughput sequencing technologies (16S rRNA gene sequencing, metagenomics, and metatranscriptomics) and mass spectrometry/nuclear magnetic resonance-based omics approaches (metabolomics and proteomics) facilitate in-depth analysis of the GM from microbial compositional profiling to functional characterization (Liu et al., 2025). Artificial intelligence (AI) and machine learning (ML) algorithms can integrate multidimensional data from medical imaging, clinical records, and omics studies to construct predictive models for precision diagnosis and personalized treatment (Li et al., 2023; An et al., 2025; Li et al., 2024). More importantly, GM-based interventions—such as FMT, probiotic supplementation, and targeted metabolite administration—have been proven safe and feasible in the treatment of inflammatory bowel disease and cancer immunotherapy, providing a novel therapeutic avenue for adjunctive or alternative TB treatment (Chai et al., 2025; Liu et al., 2025).

This review centers on the core question of how the GM reshapes TB diagnosis and treatment. It systematically summarizes the latest advances in three key technological fields: next-generation sequencing (NGS) and bioinformatics, metabolomics and proteomics, and AI. Additionally, it evaluates the translational potential of GM-targeted intervention strategies for tuberculosis prevention and control, delves into the controversial research findings and knowledge gaps in GM-TB-related studies, and analyze the intervention challenges and translational application barriers of FMT and probiotics in the TB field. We aim to provide a comprehensive GM-centric roadmap for TB researchers and clinicians, promoting the shift from a one-size-fits-all treatment model to an era of personalized medicine for TB.

## 2 Microbiome panoramic analysis: from sequencing to ecology

A precise characterization of the GM’s structure, function, and dynamic changes is a prerequisite for investigating the mechanisms underlying GM-TB interactions and developing targeted intervention strategies. Over the past decade, iterative innovations in sequencing technologies, the refinement of bioinformatics algorithms, and the rapid development of open-access databases have enabled researchers to move from simply identifying microbial taxa (what microbes are present) to elucidating their functional activities and host interactions (what they do and how they interact with the host). This section reviews the advantages and limitations of 16S rRNA gene sequencing, metagenomics, and metatranscriptomics; outlines the standardized analytical workflow from raw sequencing reads to ecological function inference; discusses strategies for mitigating batch effects; and summarizes the core public databases and analytical tools currently available. These contents lay a solid technical and data foundation for multi-omics integration and clinical translation in subsequent sections of this review.

### 2.1 Sequencing technology evolution: strengths and limitations of 16S rRNA, metagenomics, and metatranscriptomics

NGS has revolutionized microbiome research by providing powerful tools for comprehensive microbial community analysis. Illumina sequencing, shotgun metagenomics, and amplicon sequencing each possess distinct advantages. Illumina sequencing offers high accuracy and scalability, making it the gold standard for microbial diversity analysis (Szoboszlay et al., 2023); shotgun metagenomics enables whole-genome sequencing of all microbial taxa in a sample, thereby revealing their compositional and functional potential (Quince et al., 2017).Amplicon sequencing, particularly 16S rRNA gene sequencing, allows for targeted, high-throughput identification of bacterial and archaeal taxa (Hugerth and Andersson, 2017). NGS supports both taxonomic and functional profiling of the microbiome: amplicon sequencing facilitates the analysis of microbial community dynamics via relative abundance data, while shotgun metagenomics deciphers key metabolic pathways and ecological roles of the GM (Szoboszlay et al., 2023). Knight et al. emphasize that rigorous sampling protocols, strict quality control, and robust statistical analyses are essential for reliable interpretation of microbiome sequencing data (Knight et al., 2018).

#### 2.1.1 16S rRNA gene sequencing

16S rRNA gene sequencing is the most widely used technique for microbial community profiling (Johnson et al., 2019). It targets the highly conserved 16S rRNA gene present in all bacteria and archaea, enabling taxonomic identification down to the genus or species level (Hugerth and Andersson, 2017). This method is cost-effective and generates high-resolution taxonomic information. However, it has inherent limitations, including the inability to capture functional information of the microbial community and potential biases introduced by primer selection and sequencing errors (**Table 1**).

**Table 1:** Comparison of next-generation sequencing technologies for microbiome analysis.

Technology	Principle	Platform	Strengths	Limitations
16S rRNA gene sequencing	Targets the 16S rRNA gene, which is conserved across bacteria and archaea	Illumina (MiSeq, NovaSeq)	Cost-effective	Limited to bacteria and archaea
Shotgun metagenomics	Sequences all DNA in a sample, providing a comprehensive view of the microbial community	Illumina (NovaSeq, HiSeq)	Comprehensive taxonomic and functional profiling	More expensive
Metatranscriptomics	Sequences RNA transcripts to identify active microbial community and functional activities	Illumina (NovaSeq, HiSeq)	Identifies active microbial community	More complex and expensive

#### 2.1.2 Shotgun metagenomics

Shotgun metagenomics involves the random sequencing of all DNA present in a sample, providing a comprehensive view of the entire microbial community—including bacteria, archaea, and viruses. This technique enables the identification of microbial species, characterization of their functional potential, and detection of antibiotic resistance genes. Nevertheless, it is more expensive and computationally intensive compared with 16S rRNA gene sequencing (**Table 1**).

#### 2.1.3 Metatranscriptomics

Metatranscriptomics focuses on the sequencing of all RNA transcripts in a sample, providing insights into the active microbial community and their *in-situ* functional activities. This technique can identify which microbial genes are expressed and their expression levels, offering a dynamic snapshot of microbial metabolic activity. However, it is technically more complex and costly, requiring specialized protocols for RNA extraction, enrichment, and sequencing (**Table 1**). Each sequencing technology has unique strengths and limitations, and the selection of an appropriate method depends on the specific research question and available resources. The integration of data from multiple sequencing approaches can provide a more comprehensive and in-depth understanding of the GM and its complex interactions with the host.

### 2.2 Bioinformatics workflows: quality control (DADA2), taxonomic and functional annotation (QIIME2, MetaPhlAn 4, HUMAnN)

High-throughput sequencing data must be processed through rigorously standardized bioinformatics pipelines to convert raw reads into biologically meaningful insights. This section summarizes the current mainstream bioinformatics workflow for microbiome analysis, with a focus on three key steps: denoising and quality control, taxonomic profiling, and functional reconstruction.

#### 2.2.1 Quality control and denoising with DADA2

Callahan et al. developed Divisive Amplicon Denoising Algorithm2 (DADA2), an R package that replaces traditional operational taxonomic unit (OTU) clustering with amplicon sequence variant (ASV) identification (Callahan et al., 2016). By modeling Illumina sequencing errors, DADA2 achieves single-nucleotide resolution in microbial profiling, thereby reducing spurious microbial diversity and improving the reproducibility of results across different studies. The DADA2 pipeline integrates five key steps: (i) filtering and trimming of raw reads based on quality scores, (ii) error rate learning from each individual sequencing run, (iii) dereplication and denoising of sequencing reads, (iv) chimera removal, and (v) read-pair merging. This pipeline generates high-resolution microbial profiles that are directly comparable between samples and across different laboratories.

#### 2.2.2 Taxonomic classification via QIIME2 and MetaPhlAn 4

QIIME2 (Quantitative Insights Into Microbial Ecology 2) provides an extensible, plugin-based computational environment for end-to-end microbiome analysis (Bolyen et al., 2019). Within the QIIME2 framework, the q2-dada2 plugin performs sequence denoising, while the q2-feature-classifier plugin implements naïve Bayes or consensus-based taxonomic assignment against curated reference databases such as SILVA and Greengenes2. For shotgun metagenomic data, MetaPhlAn 4 leverages clade-specific marker genes (~1 million unique markers across more than 17,000 microbial species) to generate rapid, high-resolution taxonomic profiles without the need for whole-genome assembly (Blanco-Míguez et al., 2023). MetaPhlAn 4’s ability to resolve microbial composition at the species and strain levels makes it particularly suitable for GM-TB research, where subtle compositional shifts in the GM may exert a profound impact on host immunity.

#### 2.2.3 Functional reconstruction using HUMAnN

The HMP Unified Metabolic Analysis Network (HUMAnN) is a specialized tool for reconstructing microbial pathway and gene-family abundance from either assembled metagenomic contigs or quality-filtered sequencing reads (Suárez Araujo et al., 2009). HUMAnN first aligns sequencing reads to the ChocoPhlAn pangenome database for organism-specific gene detection; it then performs a translated search against the UniRef database for unmapped reads; finally, it maps all identified genes to MetaCyc metabolic pathways. This dual-tier approach enables the quantitative characterization of metabolically relevant microbial modules—such as SCFA biosynthesis, bile acid transformation, and tryptophan catabolism—pathways that have been repeatedly implicated in TB susceptibility and disease progression.

### 2.3 Data normalization and batch effects: ComBat, Limma, and emerging solutions

A major challenge in large-scale microbiome research is the standardization and normalization of sequencing data to ensure the reliability and comparability of research findings. Variability in experimental protocols, sequencing platforms, and data preprocessing methods across different studies can lead to inconsistent data quality and introduce systematic biases, commonly referred to as batch effects. Traditional statistical tools such as ComBat (Johnson et al., 2007) and Limma (Smyth, 2005), initially developed for microarray data analysis, are often insufficient to effectively mitigate these biases in sparse, high-dimensional microbiome datasets. This limitation highlights the urgent need for specialized bioinformatics tools and algorithms tailored to the unique characteristics of microbiome data.

To address this issue, emerging analytical frameworks have been proposed to harmonize multi-omics data and reduce batch effects. Following the initial alignment and mapping of genomic, transcriptomic, and proteomic data, normalization and scaling techniques—such as z-score normalization and quantile normalization—are applied to adjust data from different omics layers to a common scale. These procedures help mitigate batch effects and technical variability, ensuring that integrated multi-omics data reflect true biological variation rather than experimental artifacts. Additionally, dimensionality reduction methods, including Principal Component Analysis (PCA) and t-Distributed Stochastic Neighbor Embedding (t-SNE), are used to further reduce data complexity and highlight key biological patterns.

ComBat and Limma provide foundational frameworks for batch effect correction in microbiome research, while newer normalization and multi-omics integration pipelines—integrated within platforms such as MultiQC, MixOmics, and Multi-Omics Factor Analysis (MOFA) (**Table 2**)—continue to advance the field toward robust, reproducible microbiome analyses in GM-TB research.

**Table 2:** Overview of multi-omic data preprocessing and integration.

Name	Function/application area	Key features
MultiQC	Data aggregation and reporting	Aggregates results from various bioinformatics analyses into a single report, facilitating comprehensive multi-omics data visualization.
MetaboAnalyst	Multi-omics data integration and analysis	Provides tools for metabolomic data analysis, including normalization, statistical testing, and pathway analysis, integrated with other omics data.
iCluster	Integrated clustering of multi-omics data	Enables simultaneous clustering of multiple omics datasets, identifying shared patterns and associations across different molecular layers.
MixOmics	Multi-omics data integration and visualization	Offers a suite of statistical methods for integrating and visualizing relationships between different omics datasets, including PLS-DA and correlation analysis.
MOFA	Dimensionality reduction for multi-omics data	Implements factor analysis to identify latent factors that capture shared and unique variations across multiple omics layers, enhancing data interpretation.
OmicsNet	Network-based integration of multi-omics data	Constructs interaction networks that integrate genomic, transcriptomic, and proteomic data, highlighting key nodes and pathways involved in biological processes.
Cytoscape	Network visualization and analysis	Facilitates the visualization and analysis of complex interaction networks, integrating multi-omics data to identify functional relationships and network hubs.
Galaxy	Integrative omics pipelines	An open-source platform that supports the construction of custom workflows for integrating and analyzing multi-omics data using a wide range of bioinformatics tools.
MG-RAST	Metagenomic data integration	Provides a comprehensive pipeline for the analysis and integration of metagenomic data, including functional annotation and taxonomic classification across multiple datasets.
NetMoss	Network module structure shift analysis	Evaluates shifts in network modules across different states using integrated multi-omics data, identifying key bacteria and interactions associated with disease transitions.
WGCNA (Weighted Gene Co-expression Network Analysis)	Network construction and module detection	Constructs weighted gene co-expression networks, facilitating the identification of modules and hub genes across multi-omics datasets.

### 2.4 Public databases and analysis platforms

The advancement of GM-TB research is heavily dependent on robust public databases and user-friendly analytical platforms that provide high-quality reference data, secure data storage, and powerful computational tools. These resources are indispensable for benchmarking study findings, integrating multi-omics data, and facilitating hypothesis-driven research (**Table 3**). A key resource is the Human Microbiome Project (HMP) database (Turnbaugh et al., 2007), which serves as a foundational reference by cataloging microbial communities across various human body sites in healthy individuals, enabling researchers to contextualize and compare TB-associated GM dysbiosis. For large-scale metagenomic data storage and analysis, the Integrated Microbial Genomes & Microbiomes (IMG/M) system offers a vast repository of curated metagenomic datasets (Markowitz et al., 2012; Chen et al., 2019), supporting comparative analysis of microbial functions and communities relevant to TB.

**Table 3:** Resources, databases, and platform for GM-TB research.

Name	Function/application area	Key features
Human microbiome project (HMP)	Microbiome database	Comprehensive data on human microbiota across various body sites, serving as a reference for comparative studies.
Integrated microbial genomes & microbiomes (IMG/M)	Microbiome database	Repository for metagenomic datasets, enabling access and analysis of microbiome data relevant to TB studies.
TB database	TB-specific database	Curated genomic, transcriptomic, and proteomic data specific to *MTB* and related strains.
Mycobrowser	TB-specific database	Platform for exploring genomic and functional annotations of Mycobacterium species, facilitating host-microbe studies.
OmicsDI	Multi-omics integration platform	Integrates various omics datasets, allowing comprehensive analyses involving genomics, transcriptomics, proteomics, etc.
MetaboAnalyst	Multi-omics integration platform	Supports metabolomics data analysis and integrates with other omics data types for holistic metabolic interaction studies.
KEGG (Kyoto Encyclopedia of Genes and Genomes)	Functional annotation tool	Provides pathway maps and functional annotations to understand metabolic and signaling pathways in GM-TB interactions.
DeepKEGG	Functional annotation tool	Integrates genomic, transcriptomic and epigenomic features in a pathway-centric neural architecture
SEED	Functional annotation tool	Framework for annotating and analyzing genomic and metagenomic data, exploring functional capabilities of microbial communities.

For TB pathogen-specific research, specialized databases are available. The TB Database (TBdb) (Reddy et al., 2009; Galagan et al., 2010) provides a comprehensive collection of curated genomic, transcriptomic, and proteomic data for MTB and related mycobacterial strains, facilitating in-depth studies of pathogen biology and host-pathogen interactions. Complementarily, Mycobrowser offers a specialized platform for exploring the genomic and functional annotations of *Mycobacterium species*, aiding in the identification of virulence factors, potential drug targets, and vaccine candidates (Kapopoulou et al., 2011; Gong et al., 2021; Jiang et al., 2023b; Jiang et al., 2023a; Peng et al., 2024; Wang et al., 2024; Zhang et al., 2025; Tian et al., 2026).

Beyond data repositories, powerful analytical platforms are crucial for translating raw sequencing data into biological insights. Galaxy is an open-source, web-based platform that democratizes bioinformatics research by allowing researchers without extensive programming expertise to construct, share, and execute reproducible computational workflows for multi-omics data integration and analysis (Ko et al., 2012; Heo et al., 2013; Galaxy, 2024). For metabolomic and microbiome-specific analysis, MicrobiomeAnalyst provides a user-friendly interface that supports comprehensive statistical analysis, data visualization, and integration of microbiome data with metabolomic profiles. This platform enables researchers to identify differentially abundant microbial taxa, perform functional prediction, and uncover metabolic signatures associated with TB progression and treatment response (Dhariwal et al., 2017; Lu et al., 2023). Together, these databases and platforms form an essential cyber-infrastructure that supports the entire GM-TB research pipeline—from data acquisition and management to integrated analysis and biological interpretation.

Through the hierarchical implementation of NGS technologies, standardized bioinformatic analysis pipelines, and targeted strategies for batch effect correction, a comprehensive technical framework for the holistic profiling of the GM has been developed. This framework integrates extensive public databases and analytical platforms, thereby enabling precise analyses that span from microbial species identification to functional characterization, as well as from single-omics interpretation to multi-omics integration. Such an approach facilitates the systematic screening of microbial targets and regulatory pathways pertinent to TB intervention.

## 3 Host–microbe interactions through the lens of metabolism and proteomics

The core issue of GM–TB interaction lies in the question: “How do these microorganisms affect the host?” Integrating metabolomic and proteomic datasets allows us to dissect the molecular crosstalk between the gut microbiota and MTB, and to identify non-invasive biomarkers as well as therapeutic targets. This section focuses on the key metabolic pathways and protein networks that orchestrate the gut-lung axis in TB.

### 3.1 Metabolomics: roles of SCFAs, bile acids, and tryptophan pathways in the gut-lung axis

**Figure 1:**
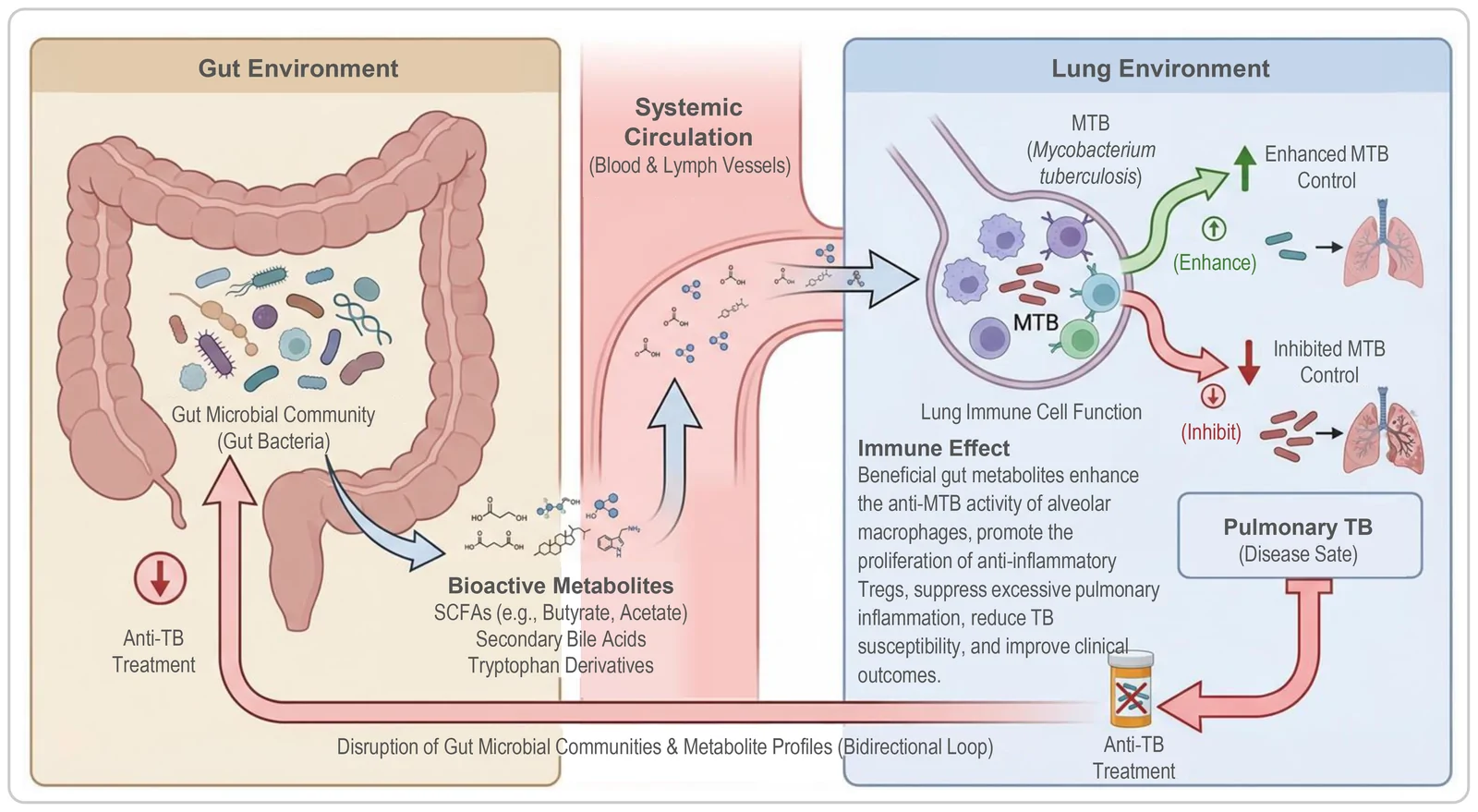
Bidirectional regulatory mechanism of the gut–lung metabolic axis in TB. Gut bacteria produce bioactive metabolites (including SCFAs, secondary bile acids, and tryptophan derivatives) that enter the systemic circulation and modulate immune cell function in the lung. These metabolites can either enhance or inhibit the host’s ability to control MTB infection. Conversely, pulmonary TB and anti-TB treatment disrupt gut microbial communities and their metabolite profiles, completing a bidirectional gut-lung regulatory loop.

The gut-lung axis concept posits that the GM influences respiratory health through bidirectional immune and metabolic crosstalk (Frati et al., 2018). In TB, the gut-lung axis is a key regulator of disease susceptibility and clinical outcomes (Osei Sekyere et al., 2020). Numerous studies have reported that active pulmonary TB is associated with distinct GM dysbiosis: beneficial commensal taxa such as *Bifidobacterium* and *Lactobacillus* are depleted, while potentially pro-inflammatory taxa—including *Proteobacteria* and *Enterobacteriaceae*—are enriched (Chai et al., 2025; Liu et al., 2025). This microbial imbalance can impair intestinal mucosal barrier integrity and skew the host immune tone toward a pro-inflammatory state. The gut microbiota produces a diverse array of metabolites—such as SCFAs, secondary bile acids, and amino acid derivatives—that circulate systemically and act as key immunomodulators. For instance, butyrate and propionate, the major SCFAs produced by microbial fermentation of dietary fiber, enhance the function of regulatory T cells and anti-inflammatory macrophages (Durán-Castañeda et al., 2024). In the lung, these metabolites can boost the antimicrobial activity of alveolar macrophages and temper excessive inflammatory responses. Conversely, TB infection can perturb the gut microenvironment via systemic inflammation, and anti-TB therapy can further disrupt the GM, forming a bidirectional feedback loop. **Figure 1** illustrates a conceptual model of the gut-lung metabolic axis in TB, where gut-derived metabolites modulate pulmonary immunity, while pulmonary TB and its treatment alter the gut microbiota and its metabolomic profile.

Key gut-derived metabolites implicated in TB include:

SCFAs: Butyrate, acetate, and propionate enhance intestinal mucosal barrier integrity and anti-inflammatory signaling via G-protein-coupled receptors (e.g., GPR43) and epigenetic modulation (e.g., histone deacetylase inhibition) (Salvi and Cowles, 2021). In TB, SCFAs can boost macrophage phagocytosis of MTB and the production of antimicrobial peptides. Reduced fecal SCFA levels have been correlated with more severe TB disease, highlighting their protective role in TB immunity.

Secondary bile acids: Gut bacteria play a pivotal role in converting host-derived primary bile acids into secondary bile acids (e.g., deoxycholic acid and lithocholic acid) (Ramírez-Pérez et al., 2017). These secondary bile acids are bioactive molecules that interact with host nuclear and cell surface receptors, including the Farnesoid X receptor (FXR) and G protein-coupled bile acid receptor (TGR5) (Chiang and Ferrell, 2020). Activation of these receptors modulates a range of physiological processes, including metabolism, inflammation, and immune responses, thus linking gut microbial bile acid metabolism to systemic host regulation. Secondary bile acids influence immune cell differentiation and may alter MTB survival in the host (Munansangu et al., 2022): certain bile acid profiles can dampen pro-inflammatory responses, potentially facilitating MTB persistence, while others can activate macrophage antimicrobial function.

Tryptophan metabolites: Gut microbes metabolize dietary tryptophan into indoles, kynurenine, and serotonin, which signal through the aryl hydrocarbon receptor (AhR) and other metabolic pathways (Keszthelyi et al., 2009). AhR activation by microbial indoles typically promotes mucosal barrier immunity and the production of interleukin-22 (IL-22). In TB, systemic tryptophan levels decline due to enhanced catabolism by both the host and microbes, while the immunosuppressive metabolite kynurenine accumulates—a phenomenon termed the “tryptophan sink”—which MTB exploits to evade host immune surveillance.

Other amino acid derivatives: Metabolites such as polyamines (e.g., spermidine) and microbial-derived amino compounds can modulate macrophage autophagy and activation (Lian et al., 2022). Perturbations in these pathways may shift the balance between host containment of MTB and bacterial dissemination.

These metabolites and their regulatory pathways form a complex biochemical dialogue between the gut microbiota and the host immune system, exerting a profound impact on TB pathogenesis. **Table 4** summarizes the major GM-derived metabolites and their known or putative effects on TB immunity.

**Table 4:** Key GM-derived metabolites influencing host immune responses relevant to TB.

Metabolite/group	Gut microbial source	Effect on TB immunity	Reference
Butyrate	Anaerobic fermenters (e.g. *Faecalibacterium*, *Roseburia*)	Enhances macrophage phagocytosis and antimicrobial activity; promotes regulatory T-cell induction (anti-inflammatory)	(Schulthess et al., 2019; Salvi and Cowles, 2021)
Acetate	Various fermenters	Modulates inflammation; provides energy substrate for host cells	(Schulthess et al., 2019)
Propionate	*Bacteroides*, *Veillonella*, others	Activates immune receptors (e.g. GPR41) on macrophages; influences chemokine production	(Haghikia et al., 2022)
Secondary bile acids (e.g. deoxycholate, lithocholate)	*Clostridium* clusters, *Bacteroides* spp. convert primary bile acids	Engage FXR/TGR5 pathways; modulate dendritic cell and T-cell responses; may alter MTB virulence	(Hofmann, 2004)
Indole and derivatives (AhR ligands)	*Clostridium*, *Bacteroides*, *Peptostreptococcus*	Activate AhR in mucosal immune cells; promote IL-22 and IL-17 responses; maintain barrier	(Dvořák et al., 2021)
Kynurenine (via host IDO/TDO activity, but amplified by microbes)	Host and gut microbes (e.g. *Lactobacillus*)	Inhibits T-cell proliferation; skews toward regulatory phenotype; high levels linked to TB progression	(Zhang et al., 2024)
Polyamines (e.g. spermidine)	*Escherichia*, *Lactobacillus*, and others	Promote macrophage autophagy and anti-MTB responses; can also support bacterial growth if unregulated	(Madeo et al., 2018)

Recent studies have highlighted the profound impact of MTB infection on gut microbial composition and metabolomic profiles (Liu et al., 2025). TB-infected individuals exhibit a characteristic disruption of the gut microbiota, marked by the depletion of beneficial commensals (e.g., *Lactobacillus* and *Bifidobacterium*) and the enrichment of opportunistic pathogens (Kailasapathy and Chin, 2000). These microbial shifts are accompanied by altered levels of SCFAs, bile acids, and amino acid metabolites—key regulators of host immunity and inflammation. This TB-associated gut dysbiosis can influence systemic immune responses via the gut-lung axis, thereby modulating TB progression, disease severity, and treatment response. Understanding these microbiota-metabolite interactions provides novel avenues for biomarker discovery and the development of targeted gut-based therapeutic strategies for TB management (Xi et al., 2024; Zhuang et al., 2024a).

### 3.2 Lipid and amino acid metabolism reprogramming: systemic metabolic signatures in TB patients

TB imposes significant metabolic demands on the host, and MTB has evolved sophisticated strategies to reprogram host cellular metabolism to create a favorable intracellular niche for survival and replication. Lipid metabolism is profoundly altered in TB: MTB induces the accumulation of lipid droplets in infected macrophages, exploiting host fatty acids and cholesterol as carbon sources for its own cell wall synthesis and energy storage (Lovewell et al., 2016; Simwela et al., 2025). Clinically, TB patients often exhibit elevated serum levels of cholesterol, triglycerides, and specific fatty acids (Chidambaram et al., 2021). Lipidomic analyses have consistently demonstrated that cholesterol esters, triacylglycerols, and acylcarnitines are upregulated in active TB; these lipid changes not only support MTB latent survival but also serve as distinctive biomarkers to distinguish TB from other pulmonary diseases.

Amino acid metabolism is also heavily dysregulated in TB. MTB-infected hosts show enhanced tryptophan catabolism via the indoleamine 2,3-dioxygenase (IDO) pathway, leading to the accumulation of kynurenine (Fujiwara et al., 2022; Liu et al., 2024). A high kynurenine-to-tryptophan ratio is closely correlated with TB disease progression, reflecting immune suppression and T-cell anergy. Similarly, the arginine-nitric oxide pathway is disrupted: MTB can deplete host arginine or shunt it away from nitric oxide synthesis, thereby weakening the bactericidal activity of macrophages. Glutamine and glutamate levels are also perturbed in TB, as MTB can utilize glutamine as a key nitrogen source for growth. Central carbon metabolism is rewired in MTB-infected host cells. Infected immune cells adopt a Warburg-like metabolic phenotype, characterized by increased glycolysis and lactate production even under aerobic conditions (Escoll and Buchrieser, 2018). This metabolic shift provides rapid ATP and biosynthetic precursors for host immune responses but also leads to the accumulation of lactate and succinate in the microenvironment. Altered levels of tricarboxylic acid (TCA) cycle intermediates (e.g., succinate, fumarate) have been detected in TB patients, reflecting mitochondrial metabolic shifts and the hypoxic microenvironment of TB granulomas.

These metabolic alterations generate distinct biochemical signatures in the blood, urine, and sputum of TB patients. For example, TB patients exhibit consistent elevation of specific carnitines (involved in fatty acid transport), depletion of essential amino acids (due to enhanced immunological consumption), and increased oxidative stress markers (resulting from chronic inflammation). **Table 5** outlines the representative metabolic changes observed in active TB compared with healthy individuals or those with latent TB infection (LTBI).

**Table 5:** Representative host metabolic alterations in active TB compared to healthy controls.

Metabolite/class	Trend in active TB	Biological significance	References
Cholesterol and sterols	Increased	Source of lipid for MTB cell wall; marker of host lipid dysregulation	(Lee et al., 2013)
Triglycerides	Increased	Energy storage; MTB utilizes host TGs under latency	(Maurya et al., 2018)
Acylcarnitines	Increased (various chain lengths)	Reflect altered fatty acid oxidation and transport	(McCoin et al., 2015)
Fatty acids (e.g. palmitate, oleate)	Increased	Supply carbon for MTB; indicate host lipid mobilization	(Allen and Martinez, 2020)
Tryptophan	Decreased	Catabolized by host and bacteria; depletion impairs T-cell function	(Mellor et al., 2003)
Kynurenine	Increased	Immunosuppressive metabolite; correlates with active TB	(Weiner et al., 2012)
Arginine	Decreased	Substrate for NO production; reduction suggests immune evasion	(Martí and Reith, 2021)
Lactate	Increased	Product of glycolysis; may indicate hypoxic or Warburg metabolism in infected tissues	(Escoll and Buchrieser, 2018)
Succinate	Increased	Stabilizes HIF-1α; can promote inflammation	(Mills and O’Neill, 2014)
Glutamine	Altered (often decreased)	Fuel for rapidly dividing immune cells; MTB also consumes glutamine	(Abnousian et al., 2023)
Coenzyme A variants (e.g. pantothenate)	Altered	Host cofactor for metabolism; changes reflect MTB manipulation	(Keen, 2021)

These host metabolic shifts are closely intertwined with gut microbial activity: elevated host fatty acids can alter gut microbial fermentation, while tryptophan depletion and kynurenine accumulation can modulate intestinal immune tone and GM composition (Gao et al., 2018). Conversely, the GM contributes key metabolites (as summarized in **Table 4**) that modify these host metabolic pathways. This intricate crosstalk highlights the potential of metabolomic profiling to map the complex host-microbe metabolic network in TB.

### 3.3 Proteomics: the tripartite protein landscape of host–pathogen–microbiota interactions

Proteomics provides a powerful framework for deciphering the complex protein-mediated interactions between the host, MTB, and the GM by characterizing the complete set of proteins expressed in a biological system. This approach captures the functional executors of biological processes, offering a dynamic snapshot of infection and host immune responses that cannot be obtained by genomic or transcriptomic methods alone.

A key mechanism of MTB pathogenesis involves the disruption of host cellular integrity to evade immune surveillance (Jiang et al., 2023c; Zhuang et al., 2023). Specific MTB antigens—including ESAT-6, 19 kDa lipoprotein, Hip1, and Hsp70—target core host organelles such as mitochondria, the endoplasmic reticulum, and phagosomes, inducing endoplasmic reticulum stress and delaying both innate and adaptive immune responses (Ghoshal et al., 2024). In response to MTB infection, the host secretes exosomes that act as double-edged messengers in immune signaling: these extracellular vesicles can carry both host antimicrobial molecules and MTB-derived proteins. Proteomic profiling of exosomes from MTB-infected macrophages has identified more than 350 human proteins, a significant proportion of which are uniquely enriched during infection, highlighting the dual role of exosomes in TB pathogenesis and immune modulation (Diaz et al., 2016).

Cellular proteomics has revealed extensive remodeling of the host proteome during MTB infection. Quantitative proteomic techniques such as Stable Isotope Labeling by Amino Acids in Cell Culture (SILAC) (Hoedt et al., 2019) and Tandem Mass Tag (TMT) (Muñoz-Prieto et al., 2021) labeling enable precise monitoring of these dynamic changes. These methods have been instrumental in uncovering infection-driven alterations—including widespread changes to the host ubiquitylome and cell-type-specific immune responses—providing essential insights into how MTB manipulates host cell biology to promote its intracellular survival (Thompson et al., 2003; Porras-Yakushi and Hess, 2014; Carvalho et al., 2020). Metaproteomic analysis, which involves protein extraction, enzymatic digestion, peptide separation by chromatography, mass spectrometric analysis, and database matching, is central to these discoveries. However, it faces significant challenges, including high false discovery rates due to shared peptide sequences across different species and the substantial computational demands of analyzing large-scale metaproteomic datasets (Ankney et al., 2018; Muselius et al., 2020).

Beyond elucidating the molecular mechanisms of TB infection, proteomics plays a vital role in advancing TB therapeutics. Comparative in silico analyses of host and MTB metabolic pathways have identified 67 potential MTB-specific drug targets, providing a rational basis for novel drug design (Anishetty et al., 2005). Furthermore, proteomic profiling has clarified the mechanisms of action of existing anti-TB drugs: rifampicin exposure downregulates key proteins involved in MTB cell wall synthesis (e.g., Ino1, FabD, EsxK, PPE60), while sulfamethoxazole induces oxidative stress and modulates the MTB electron transport chain. These findings have uncovered novel secondary modes of action of anti-TB drugs and their potential for synergistic combination therapy (Meneguello et al., 2020).

Finally, proteomics illuminates the vital function of the GM in modulating anti-TB drug efficacy. Gut microbial enzymes can modify the chemical structure of pharmaceutical compounds, thereby influencing their pharmacodynamic properties. Microbiome-directed therapies such as FMT—already established as a gold standard treatment for recurrent *Clostridioides difficile* infection—show great promise for TB by reducing the abundance of antimicrobial resistance genes and restoring a balanced GM. FMT and probiotic supplementation may enhance anti-TB therapy by reintroducing SCFA-producing taxa, reducing systemic inflammation, and strengthening host immunity—particularly in patients with drug-resistant TB (Aldrich et al., 2019; Sorbara and Pamer, 2022; Boicean et al., 2023).

### 3.4 Exosomal proteins and microbial enzymes: potential non-invasive biomarkers

Exosomes—nano-sized extracellular vesicles secreted by both host cells and pathogens—carry a diverse molecular cargo, including proteins, nucleic acids, lipids, and microbial enzymes. In recent years, these vesicles have emerged as a promising source of non-invasive biomarkers for infectious diseases, particularly TB (Li et al., 2023a). The unique composition of exosomes reflects the physiological state of their parent cells and the nature of host-pathogen interactions, making them a valuable diagnostic and prognostic tool for TB. Exosomal proteins and microbial enzymes provide primary insights into MTB biology, offering disease-specific molecular signatures that can be detected in easily accessible body fluids such as blood, saliva, and urine. Proteomic studies have demonstrated that exosomes derived from MTB-infected macrophages and the plasma of TB patients are enriched with bacterial antigens (e.g., ESAT-6, Ag85 complex, KatG, SodA) and host proteins (e.g., heat shock proteins, haptoglobin, vimentin) (Biadglegne et al., 2021). This protein signature reflects both host immune activation and MTB persistence: ESAT-6 and Ag85 are immunodominant MTB virulence factors involved in cell wall synthesis, KatG is a catalase-peroxidase that is essential for MTB to resist oxidative stress and to activate the anti-TB drug isoniazid. The presence of these proteins in circulating exosomes highlights their potential as diagnostic biomarkers to distinguish active TB (ATB) from LTBI. Host-derived exosomal proteins also carry important diagnostic information: heat shock proteins indicate cellular stress induced by MTB infection, while the downregulation of MHC class I or CD36 in exosomes may reflect MTB-mediated immune evasion strategies. Microbial enzymes present in exosomes represent another promising class of TB biomarkers; these enzymes are involved in bacterial metabolism, stress adaptation, and virulence regulation. For instance, catalase-peroxidase (KatG) and superoxide dismutase (SodA/SodB)—critical for MTB to neutralize reactive oxygen species in host macrophages—are consistently detected in exosomal preparations from TB-infected individuals. Similarly, enzymes involved in lipid metabolism and nitrogen utilization (e.g., glutamine synthetase (GlnA1), fatty acid synthases) are present in the exosomal proteome and may correlate with MTB intracellular survival strategies. The detection of these microbial enzymes in exosomes provides direct evidence of ongoing intracellular MTB infection, even when bacterial loads are too low to be detected by conventional smear microscopy or culture.

From a clinical perspective, exosomal proteins and microbial enzymes possess several key advantages as TB biomarkers:

Stability: The protective lipid bilayer of exosomes shields their cargo from enzymatic degradation in body fluids, ensuring high stability and detectability.

Disease specificity: Specific proteins and enzymes are upregulated in ATB but absent or downregulated in LTBI or other pulmonary diseases. For example, haptoglobin and inflammatory proteins are highly enriched in exosomes from ATB patients, while stress proteins such as HSP90 correlate with active disease states (Biadglegne et al., 2021).

High-throughput detectability: These molecules can be quantified using high-throughput analytical platforms such as mass spectrometry, ELISA, or immuno-PCR, making them compatible with routine clinical workflows.

Treatment response monitoring: Their levels dynamically change in response to anti-TB therapy, enabling the monitoring of treatment efficacy and the early detection of disease relapse (Zhao et al., 2025).

In conclusion, exosomal proteins and microbial enzymes represent robust, non-invasive biomarkers that capture the complexity of host–pathogen interactions in TB. While challenges remain—including the lack of standardized exosome isolation protocols and the need to ensure reproducibility across laboratories—the integration of exosomal proteomics into clinical diagnostics represents a groundbreaking advance in TB management. By providing accurate information on infection status, these biomarkers have the potential to not only improve early TB detection but also to guide therapeutic decisions and monitor treatment outcomes, thereby strengthening global TB control strategies (Biadglegne et al., 2021).

### 3.5 Multi-omics integration strategies: a metabolic-proteomic-genomic joint analysis framework

Multi-omics joint analysis in TB research captures upstream genetic regulation, mid-level protein function, and downstream metabolic consequences of infection. These studies integrate diverse biological data types into a single analytical model, uncovering complementary roles of metabolites, proteins, and genes in TB pathogenesis. Researchers typically apply this framework by sequencing miRNAs, quantifying metabolites via liquid chromatography-mass spectrometry (LC-MS), and measuring cytokines/chemokines using multiplex immunoassays. After individual analysis of each dataset, the three omics layers are combined in a decision-tree classifier to identify optimal biomarker panels consisting of genomic, proteomic, and metabolomic features. This integrated model has achieved outstanding performance in distinguishing TB patients from healthy controls, highlighting the power of multi-omics joint analysis (**Table 6**) (Krishnan et al., 2021).

**Table 6:** Representative biomarkers obtained from combined analysis of metabolic-proteomic-genomic.

Method/name	Antigen(s) involved	Proteomic method/platform	Key findings	References
Serum biomarker discovery in advanced HIV	Host serum (miRNAs, metabolites, cytokines)	LC-MS metabolomics, multiplex cytokine assay, NGS for miRNAs	miR-215-5p + gamma-glutamylthreonine panel achieved Area Under the Curve(AUC) 0.965 for TB diagnosis in PLWH	(Krishnan et al., 2021)
Circulating lipid biomarkers	Host plasma lipids, including PC (14:0_22:6)	Plasma lipid omics (LC-MS), integrated with immune-metabolite profiling	Lipid biosignatures distinguished TB from LTBI, NTM and ODx with AUC 0.7-0.9; PC(14:0_22:6) validated as top predictor	(Tien et al., 2024)
Mechanism of DA-CB	*M. smegmatis* lipids (mycolic acid, wax monoesters, FAs)	Untargeted LC-MS/MS (lipidomics & metabolomics), NMR, proteomics	DA-CB inhibited mycolic acid biosynthesis, perturbed pyrimidine and fatty acid metabolism, validated new anti-TB MoA	(Sakallioglu et al., 2022)
Hyper-virulent strain analysis	*M. tuberculosis* H112 starin mutations (FadE5, Rv0178, higB, pip)	RNA-seq + label-free quantitative LC-MS/MS	25 genes consistently dysregulated: linked hypervirulence to specific mutations affecting virulence regulons	(Rajwani et al., 2022)
Review of omics in TB drug discovery	Not antigen-specific, focus on M.tb pathways (folate, PDIM, ESX-1, mycolic acid)	Genomics, transcriptomics, proteomics, metabolomics, lipidomics	Omics applied to identify druggable pathways, confirm compound MoA, and explore antimicrobial resistance	(Goff et al., 2020)
TB-diabetes comorbidity	Host plasma cytokines, whole-blood transcriptome, urinary eicosanoids	RNA-seq, Luminex assays, LC-MS eicosanoid profiling	Identified multi-omic signature	(Vinhaes et al., 2024)

The fundamental principle behind multi-omics integration is the complementary nature of different biological hierarchical levels. The genome (including miRNA) reveals genetic predispositions and regulatory signals that suggest potential outcomes; the proteome reflects the current functional state, showing what is actually occurring; and the metabolome displays the final phenotypic results of past events. Combining these three omics layers into a single analytical framework allows for simultaneous observation of the same biological process across three temporal stages, greatly improving the completeness and accuracy of signal detection. Diagnosis remains the main challenge in TB prevention and control. Traditional methods like the tuberculin skin test (TST) and interferon-gamma release assay (IGRA) cannot differentiate between LTBI and ATB. The key advantage of multi-omics integration lies in identifying genuine disease-driving biomarkers through causal inference. By integrating lipidomics based on ultra-high performance liquid chromatography-tandem mass spectrometry (UPLC-MS/MS) with data-independent acquisition (DIA) proteomics, plasma samples from healthy individuals, TB patients, and cured subjects were analyzed. This approach identified three phosphatidylcholine lipids and four proteins—haptoglobin (HP), retinol-binding protein 4 (RBP4), coagulation factor XIII B subunit (F13B), and alpha-1-antitrypsin heavy chain 1 (ITIH1)—as potential biomarkers. A multi-omics random forest model built on these biomarkers showed excellent performance. Gene Set Variation Analysis (GSVA) further indicated that these molecules participate in iron homeostasis and immune response pathways (Zhu et al., 2026). This directly highlights the crucial mechanism of GM-TB interaction: gut microbiota influences host susceptibility to MTB through iron metabolism. TB patients face risks of treatment failure and relapse, and early identification of high-risk individuals remains a significant clinical challenge. Multi-omics integration combines clinical phenotypes with molecular signatures to develop highly accurate predictive models. Researchers combined clinical data from 467 TB patients with transcriptomic data from three independent public cohorts (GSE19491, GSE31312, GSE83456), encompassing 3,240 differentially expressed genes. A comprehensive evaluation of 12 machine learning algorithms found that an Extra Trees-based ensemble model delivered the best performance (Li et al., 2026). The pathways enriched in this study were mainly related to immune response and metabolism, aligning closely with the core mechanism of GM-TB interaction. This suggests that gut microbial metabolites, such as short-chain fatty acids, can regulate the lung immune environment via the gut-lung axis, thereby influencing the host’s ability to clear MTB. Adding metagenomic data to this ensemble model can further enhance the understanding of treatment responses.

**Figure 2:**
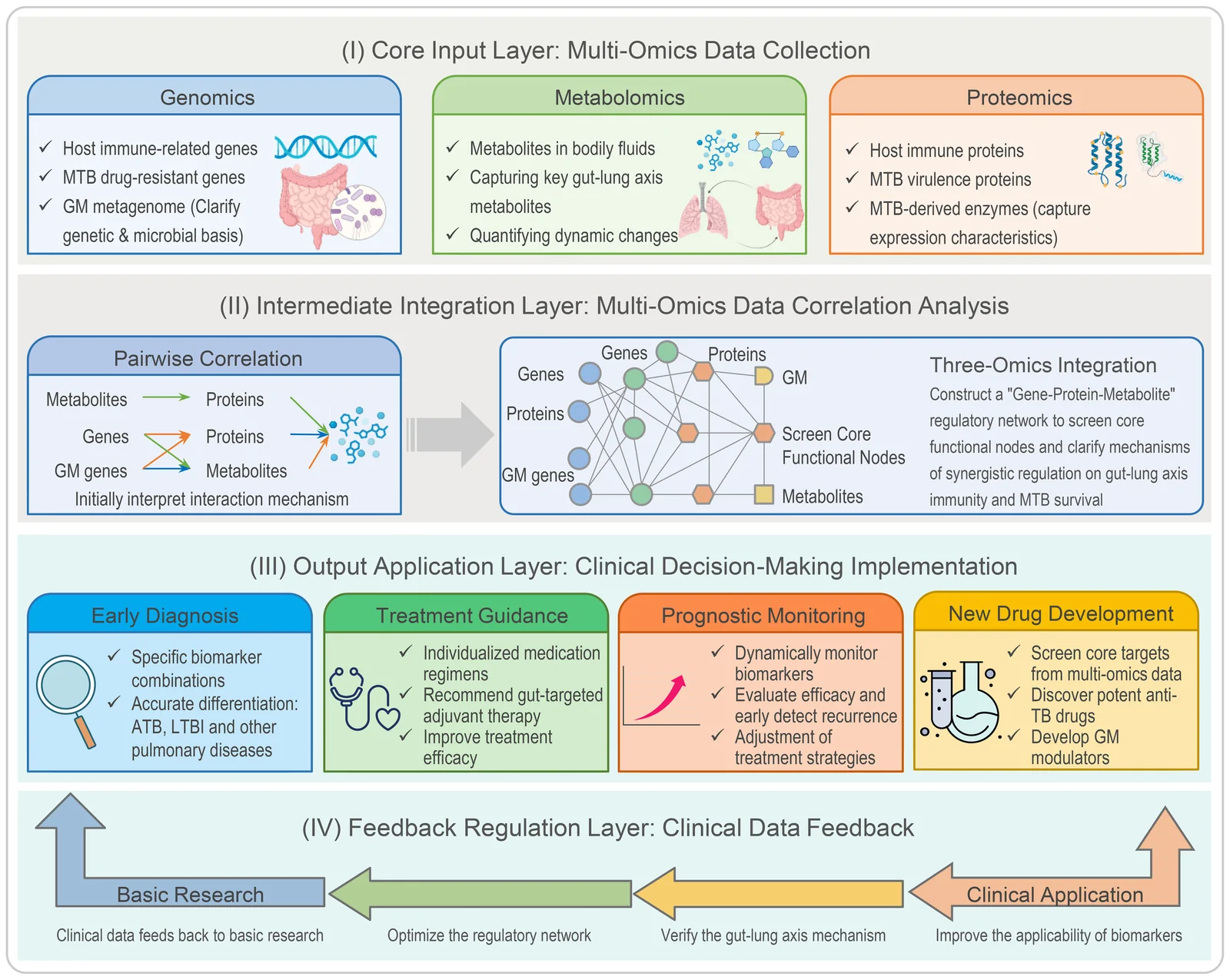
A four-layer framework for multi-omics integration to decipher GM-TB interactions and its clinical translation. (I) Core Input Layer: collection of multi-omics data including genomics, metabolomics, and proteomics; (II) Intermediate Integration Layer: correlation analysis and regulatory network construction to identify core functional nodes; (III) Output Application Layer: clinical decision-making implementation encompassing early diagnosis, treatment guidance, prognostic monitoring, and new drug development; (IV) Feedback Regulation Layer: clinical data feedback to optimize the regulatory network and improve biomarker applicability.

The integration of metabolome, proteome, and genome data is transforming clinical decision-making in TB. This approach has shown translational clinical value in various contexts, including differential diagnosis (through causal biomarker identification using Mendelian randomization), prediction of treatment response (via integrated models combining clinical and transcriptomic data), and detection of drug resistance (using multi-tool ensemble learning). For the complex, systems biology-focused issue of GM-TB interaction, multi-omics integration offers a practical route from merely describing correlations to actively intervening in causal mechanisms. **Figure 2** illustrates the mechanism of multi-omics integration in deciphering GM-TB interaction and its clinical application.

Metabolomics and proteomics have revealed the core regulatory mechanisms of the gut-lung axis in the immune regulation of TB at the molecular level, clarified the roles of key metabolites, as well as biomarkers such as exosomal proteins and microbial enzymes, and provided molecular targets and theoretical basis for the development of TB intervention measures based on the GM. However, current studies have limitations: insufficient verification of the causal mechanisms within the gut-lung axis, lack of rigorous intervention experiments such as microbiota transplantation and metabolite supplementation; low detection rate and limited coverage of low-abundance gut microbial proteins and MTB intracellular proteins; the clinical application of biomarkers lags behind basic research, with a lack of multi-center and large-scale clinical verification, and insufficient specificity, making it difficult to distinguish TB from other pulmonary diseases such as NTM infection and pneumonia.

## 4 AI-driven biomarker discovery and diagnostic decision-making

AI and ML have emerged as powerful tools in microbiome research, particularly in the context of TB. These technologies enable the analysis of complex, high-dimensional datasets, facilitating the identification of novel biomarkers, the improvement of diagnostic accuracy, and the optimization of treatment strategies (An et al., 2025; Zhuang et al., 2024a; Zhuang et al., 2024b). By integrating diverse data types—including microbiome profiles, clinical metadata, and medical imaging data—AI-driven models can provide personalized insights into disease progression and therapeutic response. This section explores the application of AI and ML in microbiome data analysis, medical imaging for TB diagnosis, and the development of multi-modal models for comprehensive diagnostic and prognostic assessment in TB.

### 4.1 Machine learning in microbiome data analysis

AI and ML algorithms have become essential tools for GM data processing, pattern recognition, and functional prediction. Traditional statistical methods often struggle to analyze high-throughput, large-scale, and high-dimensional microbiome datasets, facing challenges such as the curse of dimensionality and an inability to capture nonlinear biological relationships. In contrast, AI algorithms provide powerful approaches for data analysis, interpretation, and predictive modeling (Metwaly et al., 2025). Advances in these algorithms are critical for deepening our understanding of the complex interactions between the GM and various health conditions—including TB—and for developing personalized therapeutic strategies (Ongsulee, 2017).

In TB prevention and control, ML algorithms are increasingly applied to develop risk prediction and classification models based on GM profiles, as MTB infection induces significant alterations in gut microbial composition. Chai et al. trained an RF classifier that achieved high accuracy in distinguishing TB patients from healthy individuals (Chai et al., 2024). Another study used an RF regression model to evaluate the co-variation among peripheral inflammatory pathways, microbial species abundance, and sputum MTB load, providing novel insights into TB pathogenesis and informing improved treatment strategies (Wipperman et al., 2021). Current ML models for TB risk prediction and diagnosis based on GM features are summarized in **Table 7**; these studies underscore the central role of the GM in TB and its potential application in disease monitoring and management.

**Table 7:** ML algorithms for TB risk prediction & diagnosis based on GM features.

ML algorithm	GM features	Cohort size (cases vs. controls)	Intended purpose	Key performance	Validation method	Reference
RF	Species-level abundances (shotgun metagenomics)	46 ATB vs 31 HC	ATB diagnosis	AUC 0.846 (training), 0.767 (test)	5 × 10−fold CV + independent test (16 vs 30)	(Hu et al., 2019)
RF	16S genus-level profiles	56 ATB, 36 LTBI, 50 HC	Distinguish ATB/LTBI/HC	AUC 0.82 (ATB vs HC), 0.87 (ATB vs LTBI)	5 × 10−fold CV + internal test set	(Wang et al., 2022)
RF	Mucosal 16S genus abundances	71 gut biopsies (ITB, Crohn’s, HCs)	Differentiate ITB vs Crohn’s disease	AUC ≈ 0.98	5−fold CV with feature selection	(He et al., 2021)
LASSO Logistic Regression	Fecal metabolites (LC–MS)	(Not specified)	Diagnose/predict ATB & progression	Excellent ROC discrimination	LASSO feature selection + ROC validation	(Luo et al., 2023)
Single-taxa ROC	Fecal 16S bacterial taxa (relative abundance)	19 ATB, 21 LTBI, 20 HC	Identify biomarkers for TB status	AUC 0.72–0.81 (individual taxa)	Cross-sectional ROC per taxon	(Huang et al., 2023)
RF Regression	Metagenomics + host transcriptome	35 ATB + 20 post-treatment + 55 HC	Predict inflammation recovery	Significant regression performance	Cohort follow-up + RF regression	(Wipperman et al., 2021)
RF	Blood microbiome genera via RNA−seq	73 TB vs 62 HC	Blood-based TB diagnosis	AUC 0.894	RNA−seq + RT−qPCR validation	(Gu et al., 2025)
RF/XGBoost/SVM/k−NN	Urine metabolomics	35 TB vs 19 NTM	Distinguish TB vs NTM	AUC > 0.8 (multi-algorithm)	Algorithm comparison with CV	(Anh et al., 2024)
Graph Neural Network	Phylogenetic multi-omics graph	(Not specified)	Phenotype prediction	(not specified)	Exploratory framework only	(Irwin et al., 2024)

RF, Random Forest; SVM, Support Vector Machine; XGBoost, extreme gradient boosting; k-NN, k-Nearest Neighbors; ATB, Active TB; LTBI, Latent TB infection; HC, Healthy control; ITB, Intestinal TB; NTM, Nontuberculous mycobacteria. Note: Most models achieve an AUC ≥0.8 (some near 0.9), indicating strong discriminatory power for TB-related phenotypes.

### 4.2 AI in TB diagnosis and treatment prediction

**Figure 3:**
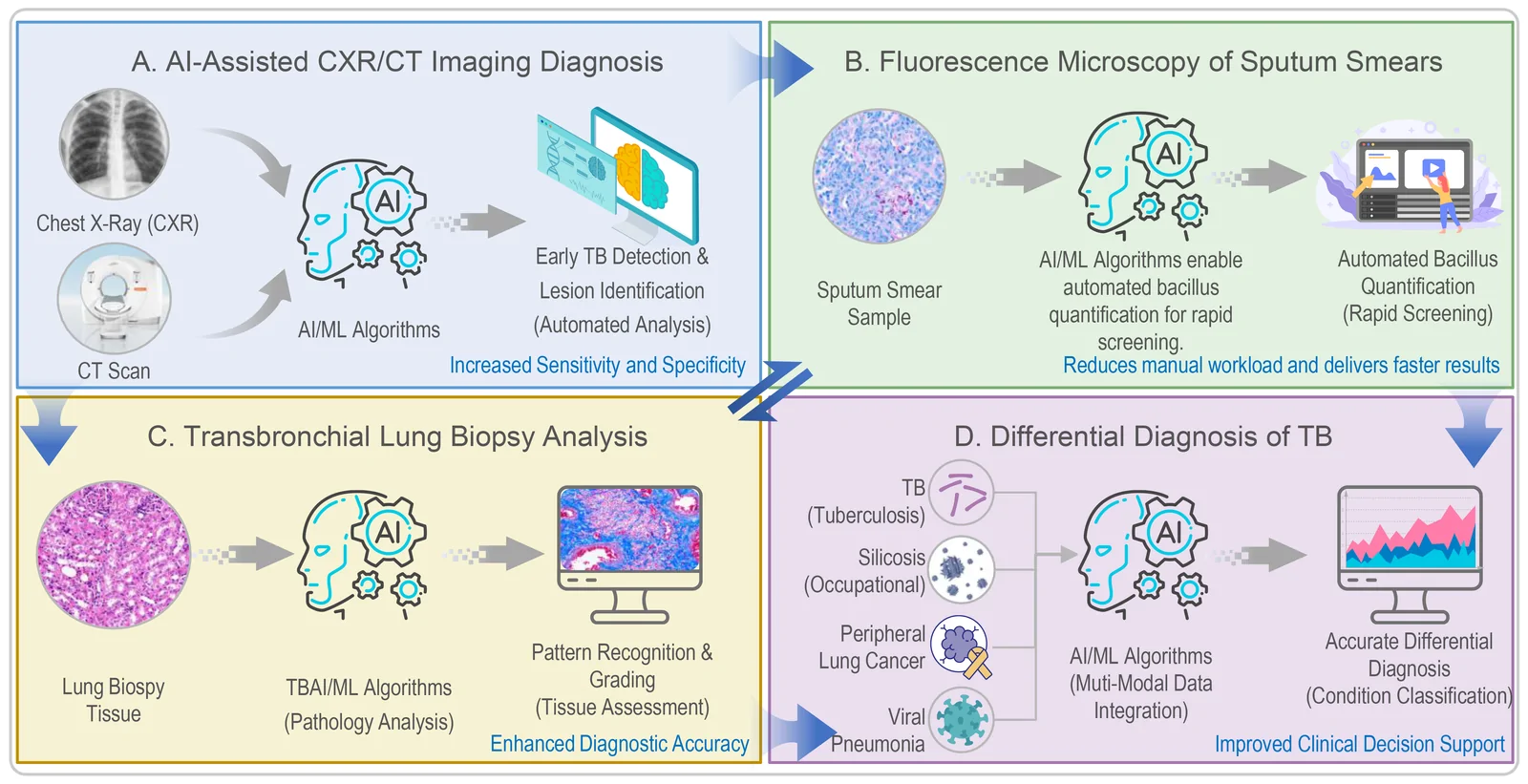
Applications of AI/ML algorithms in the early and accurate diagnosis of TB. AI/ML algorithms are applied across multiple TB diagnostic modalities, including AI-assisted CXR/CT imaging diagnosis for early TB detection and lesion identification **(A)**, fluorescence microscopy of sputum smears for automated bacillus quantification **(B)**, transbronchial lung biopsy (TBLB) analysis for pattern recognition and grading **(C)**, and differential diagnosis of TB from silicosis, peripheral lung cancer, and viral pneumonia through multi-modal data integration **(D)**.

In recent years, the rapid advancement and widespread adoption of AI have presented new opportunities for the precise and differential diagnosis of TB (**Figure 3**).

CXR remains a crucial tool for the early detection of pulmonary TB. In recent years, multiple AI-assisted software tools have been developed to support large-scale TB screening, effectively improving diagnostic efficiency and optimizing outpatient clinical workflows. Studies have shown that AI-assisted diagnostic systems outperform physician-only diagnosis across multiple metrics, including sensitivity, specificity, positive/negative predictive values, and overall diagnostic accuracy (Li et al., 2023). Compared with CXR, computed tomography (CT) is more sensitive for detecting active TB features and parenchymal abnormalities (e.g., cavitation, consolidation, centrilobular nodules) (An et al., 2025). Yan et al. developed a fully automated deep learning-based CT analysis system for the detection, diagnosis, and quantification of TB lesions, achieving a classification accuracy of 81.1% to 91.1%—comparable to that of experienced radiologists for key imaging findings (Gong et al., 2018). Li et al. utilized 3D convolutional neural networks (3D-CNNs) to construct a model capable of automatic TB lesion classification (e.g., miliary, cavitary, nodular types) and the generation of quantitative diagnostic reports (Li et al., 2021).

AI applications can help reduce misdiagnoses and false negatives caused by human error: a study on AI-assisted histopathological analysis of TBLB samples showed a significantly higher sensitivity in detecting acid-fast bacilli (AFB) compared with conventional microbiological methods (86% vs. 29%, *P* = 0.04) (Zaizen et al., 2022). These examples underscore the clinical feasibility and accuracy improvements brought by AI in TB imaging and laboratory diagnostics.

AI has also been widely applied to the differential diagnosis of TB and other pulmonary diseases. Young et al. developed an ML-based computer-aided diagnosis (CAD) model to distinguish between silicosis and TB in South African gold miners; the model was evaluated in real-world clinical settings and achieved a sensitivity of 90.1% and a specificity of 98.2%, demonstrating its practical value in improving diagnostic efficiency (Young et al., 2020). In another study, multivariate logistic regression was used to construct separate clinical, radiomic, and combined models to differentiate pulmonary adenocarcinoma nodules from tuberculomas on high-resolution CT images; the radiomic model yielded an AUC of 0.801–0.825, outperforming both the clinical and combined models (Li et al., 2024). Additionally, to address the diagnostic challenge of distinguishing pediatric pneumonia from TB in sputum-negative cases, a study applied AI algorithms and achieved a diagnostic accuracy of 97.7%—significantly higher than that of attending physicians (79.9%) (Wang et al., 2019).

Beyond diagnosis, AI also plays an important role in monitoring treatment response and predicting clinical outcomes in TB therapy. The growing prevalence of drug-resistant TB (DR-TB) has necessitated prolonged and individualized treatment regimens (Bagcchi, 2023), often involving second-line anti-TB drugs that carry a high risk of multi-organ toxicity (Gupta et al., 2020; Shah et al., 2020). Accurate and timely monitoring of treatment efficacy and prediction of clinical outcomes are essential for shortening treatment duration, reducing the burden of drug resistance, and improving patient prognosis (Rockwood et al., 2016; Goletti et al., 2018). Given its efficiency and scalability, AI has gained significant attention for its applications in assessing anti-TB drug response and predicting treatment outcomes (Asad et al., 2020; Verboven et al., 2024). One key application is predicting the optimal treatment duration to guide personalized therapy. Heyckendorf et al. developed a ML model named the “Whole-blood RNA Therapy-End Model,” which utilizes 22 gene biomarkers to predict treatment duration and outcomes in patients with drug-sensitive and extensively drug-resistant pulmonary TB (DS-GVC). The model demonstrated high predictive accuracy, with an AUC of 0.94 (95% CI: 0.90–0.98) (Heyckendorf et al., 2021). Predicting adverse drug reactions is also important for optimizing therapy. Hepatotoxicity is a common complication during second-line anti-TB treatment and may lead to treatment interruption or poor outcomes (Keshavjee et al., 2012; Song et al., 2019; Mbuh et al., 2021). Zhong et al. developed an interpretable prediction model using the XGBoost algorithm, identifying four key clinical predictors of anti-TB drug-induced liver injury (ATDH): the most recent alanine aminotransferase (ALT) value, the average rate of change in the last two ALT measurements, and cumulative doses of pyrazinamide (PZA) and ethambutol (EMB) (Zhong et al., 2021). The model achieved a precision of 90%, recall of 74%, classification accuracy of 76%, and balanced error rate of 77%, offering a valuable tool for clinicians to mitigate severe liver toxicity in TB patients. Sauer et al. analyzed TB treatment datasets using multiple ML methods, including stepwise forward selection, backward elimination, LASSO regression, random forest, and SVMs, to predict treatment failure. These models outperformed conventional statistical methods, with most achieving AUCs above 0.70; the forward selection model yielded the highest AUC of 0.74. Imaging (particularly CT features) and demographic variables were identified as key determinants of poor outcomes (Sauer et al., 2018).In addition, AI has been employed to monitor MTB drug resistance mutations during treatment (Cirillo et al., 2017) and to predict the occurrence of multidrug-resistant TB (MDR-TB) based on clinical and imaging data (Li et al., 2023b). Collectively, these studies highlight the potential of AI in treatment response monitoring and prognostic evaluation in TB care, providing more precise decision support for clinicians.

### 4.3 Future prospects and challenges of AI in personalized and precision TB treatment

The WHO currently recommends a standardized six-month treatment regimen for TB that includes a two-month intensive phase with isoniazid (INH), rifampicin (RIF), pyrazinamide (PZA), and ethambutol (ETB), followed by at least a four-month continuation phase with INH and RIF (World Health Organization, 2022). However, due to inter-individual differences in general health status, immune responses, pathogen virulence, and disease severity, the actual treatment duration required may vary significantly (Heyckendorf et al., 2021). Personalized medicine, employing individualized genotypic and phenotypic profiling to guide clinical management, enhances TB treatment outcomes by incorporating patient-specific and environmental determinants. AI are playing an increasingly transformative role in this personalization process (Holzman et al., 2018; Lange et al., 2018). In one study, researchers first identified nine drug characteristics and fourteen treatment attributes through expert consensus and literature review. Using a dataset comprising 3,895 treatment–expert feedback pairs, they trained a ML-based clinical decision support system (CDSS), which demonstrated good usability in routine care settings (Verboven et al., 2024).

Applying precision medicine to drug-resistant TB (DR-TB) holds potential to improve disease management and prevention in underserved communities by minimizing adverse effects through exclusion of potentially toxic drugs and by optimizing treatment outcomes (**Figure 3**) (Mahomed et al., 2019). AI has already been deployed in various areas of TB precision care, including image-based early diagnosis (Jaeger et al., 2018; Gong et al., 2022), accurate prediction of treatment outcomes (Srivastava et al., 2016; Mburu et al., 2018; Sauer et al., 2018), anti-TB drug design (Kouchaki et al., 2019), prediction of pathogen drug resistance (Yang et al., 2018; Drouin et al., 2016; Aytan-Aktug et al., 2020; Portelli et al., 2020), drug dosing and combination therapy optimization (Lee et al., 2017; Deshpande et al., 2018), and targeted drug delivery system development (Kouchaki et al., 2019). In clinical practice, AI assistance not only improves the efficiency and accuracy of early TB diagnosis but also enables physicians to identify risk factors for treatment failure in individual patients—such as a history of drug allergies or specific radiographic features—thereby enhancing the prediction of therapeutic response and prognosis. This facilitates personalized drug regimen recommendations tailored to different clinical scenarios (Mahomed et al., 2019).

**Figure 4:**
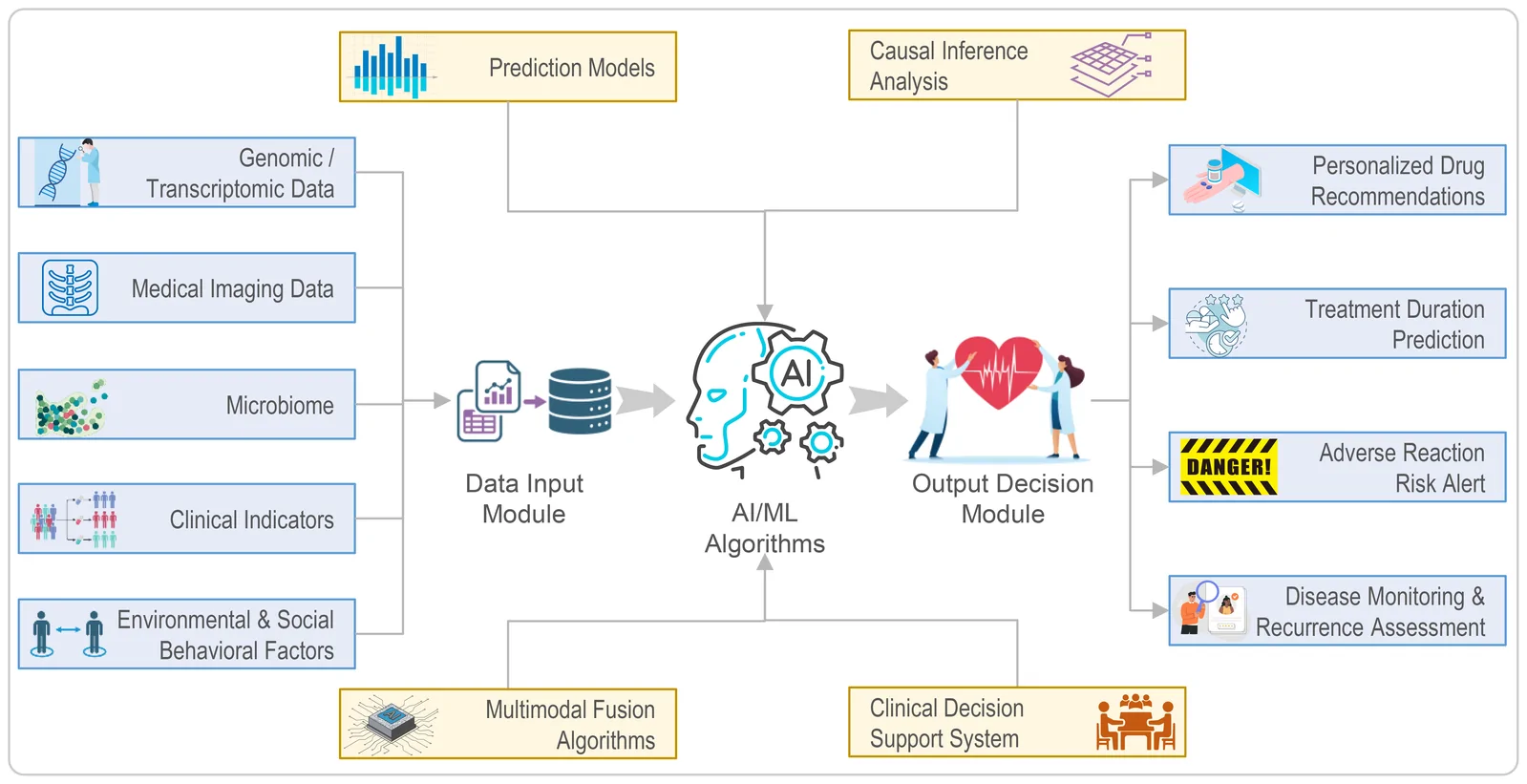
Key application aspects and process pathways of AI in precision medicine for TB. The framework integrates multi-modal data inputs (genomic/transcriptomic data, medical imaging, microbiome profiles, clinical indicators, and environmental/social/behavioral factors) through AI/ML algorithms, supported by prediction models, causal inference analysis, multimodal fusion algorithms, and clinical decision support systems, to generate personalized outputs including drug recommendations, treatment duration prediction, adverse reaction risk alerts, and disease monitoring with recurrence assessment.

The application of AI is shifting the clinical management of TB from a “one-size-fits-all” model toward individualized treatment strategies, offering valuable advancements in the field of precision medicine (**Figure 4**) (Mahomed et al., 2019). However, the clinical translation of AI in TB care still faces multiple challenges. First, the generalizability of AI models is limited by issues such as small sample sizes, high data heterogeneity, and the lack of standardized annotation and data-sharing frameworks for TB-related multimodal datasets (Kumar et al., 2025). Second, many current AI models function as “black boxes,” making their internal logic difficult for clinicians to interpret; the lack of model interpretability remains a major barrier to clinical adoption (Srinivasu et al., 2022). Additionally, AI-assisted diagnosis systems often fail to comprehensively analyze clinical symptoms and may overemphasize subtle lesions, leading to a higher risk of false positives (An et al., 2025). Infrastructure limitations further hinder AI deployment in remote and low-resource settings. The absence of high-performance computing resources and electronic health record systems impairs the implementation of AI tools. Ethical concerns also arise when integrating AI into treatment monitoring and outcome prediction. Issues such as data privacy, informed consent, and the unclear assignment of liability in the event of AI-related diagnostic or therapeutic errors must be carefully addressed (Liang et al., 2022; Cestonaro et al., 2023). Before AI algorithms can be routinely applied in TB precision care, randomized controlled trials are essential to assess whether AI-assisted strategies are superior to, equivalent to, or non-inferior to current standard-of-care treatments (He et al., 2021).

Looking ahead, AI holds significant potential in advancing personalized and precision TB care. The integration of causal inference techniques may help overcome the current reliance on correlative associations, enabling identification of true causal relationships between gut microbiota or clinical features and disease onset or treatment outcomes (Bing et al., 2022). To address ethical and regulatory challenges, secure medical data infrastructure and interoperable data governance frameworks are needed to ensure AI systems comply with healthcare standards and are prepared for risk and liability management. Multimodal AI models that integrate genomic, imaging, microbiome, and clinical data can enhance diagnostic and prognostic accuracy (Han et al., 2025). Li et al. developed a multimodal model combining CT radiomics and clinical indicators to successfully differentiate infections caused by MTB and nontuberculous mycobacteria (Li et al., 2025). Moreover, the use of large language models (LLMs), such as GPT-4, in clinical microbiology is emerging, showing early promise in medical literature synthesis, patient history summarization and decision support (Zhou et al., 2025).

AI and ML technologies have demonstrated considerable efficacy in mining and accurately analyzing complex, multidimensional datasets, including GM profiles, multi-omics data, and clinical imaging. These capabilities substantially enhance the precision of TB diagnosis and improve the prediction of treatment outcomes. Furthermore, AI and ML offer advanced analytical tools and decision support systems that facilitate the personalized design of GM-targeted intervention strategies, enable real-time monitoring of intervention effects, and predict therapeutic responses. This advancement supports the transition of microbiome interventions from broad-spectrum modulation to precise, targeted implementation. Nonetheless, several fundamental challenges persist within this domain. These include limited sample sizes and significant heterogeneity across cohorts; a lack of external validation across diverse geographic regions and populations; insufficient integration of underlying biological mechanisms, which hampers mechanistic translation; and the presence of biases and errors in diagnostic and predictive processes. Specifically, AI-based imaging systems tend to over-detect minor lesions while neglecting the comprehensive integration of clinical symptoms, resulting in potential misdiagnoses and overtreatment. Additionally, there exists a notable gap in intervention research, as microbial biomarkers identified through AI methodologies have yet to be incorporated into targeted clinical intervention trials, and the precise regulatory pathways remain unverified.

## 5 Intervention strategies based on the GM

The GM has emerged as a promising target for interventions aimed at improving TB outcomes. By modulating the GM, it is possible to enhance host immunity, reduce inflammation, and potentially improve the efficacy of existing treatments. This section explores various intervention strategies, including microbiota transplantation, probiotics, prebiotics, diet, and their integration with existing TB therapies.

### 5.1 Microbiota modulation: FMT, probiotics, prebiotics, and dietary interventions

Modulating the GM through interventions such as FMT, probiotics, prebiotics, and dietary changes holds significant potential for improving TB outcomes. These approaches aim to restore a healthy microbial balance, enhance immune function, and reduce systemic inflammation, thereby supporting the host’s ability to combat MTB infection.

#### 5.1.1 FMT

FMT involves the transfer of fecal material from a healthy donor to a recipient to restore a balanced GM. In the context of TB, FMT has been proposed as a potential therapy to restore microbial diversity and function, which are often disrupted in TB patients (Li et al., 2019; Seyed Aria and Alexander, 2024). Studies have shown that FMT can reduce the abundance of antimicrobial resistance genes and restore microbial balance, suggesting its potential as an adjunct therapy in TB management (Woodworth et al., 2023). However, safety and efficacy concerns remain, and further research is needed to fully understand the long-term effects of FMT in TB patients.

#### 5.1.2 Probiotics and prebiotics

Probiotics are live microorganisms that, when administered in adequate amounts, confer a health benefit on the host. Prebiotics are nondigestible food ingredients that beneficially affect the host by selectively stimulating the growth and activity of one or a limited number of bacteria in the colon. Both probiotics and prebiotics have been shown to enhance the efficacy of TB treatment by modulating the GM and strengthening host immunity (Fujimura et al., 2010). For example, specific probiotics such as Lactobacillus and Bifidobacterium can elevate SCFA levels, which are known to have anti-inflammatory and immune-boosting effects (Aldrich et al., 2019). Prebiotics, such as indigestible fibers, can selectively boost SCFA-producing taxa, further supporting gut health and immune function (Sorbara and Pamer, 2022; Boicean et al., 2023).

#### 5.1.3 Dietary interventions

Diet plays a key role in shaping the GM. Dietary interventions aimed at promoting a healthy GM can have a positive impact on TB outcomes. For instance, diets rich in fiber can enhance SCFA production, which is beneficial for gut health and immune function (Tanes et al., 2021). Conversely, diets high in fat or sugar can disrupt the GM and contribute to inflammation, potentially exacerbating TB symptoms (Turnbaugh et al., 2009). Therefore, dietary recommendations that promote a balanced GM can be an important component of TB management strategies.

### 5.2 Targeted metabolic pathways: SCFA precursors, bile acid receptor agonists, and IDO inhibitors

Focusing on specific metabolic pathways allows for precise adjustment of the gut microbiota, which can lead to better outcomes in TB. Precursors of SCFAs have anti-inflammatory and immune-boosting properties, encourage SCFA production, support gut health, and enhance the host’s defense against MTB. Bile acids and their receptors are key players in regulating metabolism and immunity. Agonists of bile acid receptors may be used as treatments to amplify the protective effects of SCFAs and other metabolites, showing promising potential to improve TB prognosis. Additionally, inhibitors of IDO can strengthen immune responses against MTB, serving as supplementary therapies to increase the effectiveness of current anti-TB medications and slow disease progression.

### 5.3 Vaccine potentiation: enhancing BCG and next-generation vaccines with GM

The GM can significantly influence the efficacy of vaccines by modulating immune responses. This section explores how GM modulation can enhance the immunogenicity of existing vaccines, such as Bacille Calmette-Guérin (BCG), and support the development of next-generation TB vaccines.

#### 5.3.1 BCG vaccine

The BCG vaccine is the only currently available vaccine for TB but has variable efficacy in adults (Gong e al., 2022; Yan et al., 2022; An et al., 2025). Modulating the GM to enhance BCG immunogenicity is a promising strategy for improving vaccine efficacy (Nyangahu et al., 2023; Jeyanathan et al., 2022). For example, probiotics and prebiotics can enhance the immune response to BCG vaccination by promoting the growth of beneficial bacteria that boost immune function (Mojgani et al., 2025).

#### 5.3.2 Next-generation vaccines

Next-generation TB vaccines are being developed to address the limitations of BCG. GM modulation can serve a vital function in enhancing the immunogenicity of these new vaccines. For instance, exosomes derived from MTB-infected cells can be used as vaccine platforms, and their immunogenicity can be enhanced by GM modulation (Chen, 2025). Additionally, microbiome-informed adjuvants, such as probiotics and prebiotics, can be used alongside next-generation vaccines to improve their efficacy (Jeyanathan et al., 2022).

### 5.4 Drug repurposing and combination therapies: GM-mediated efficacy and toxicity reassessment

The GM can influence the pharmacodynamics of drugs, including those used to treat TB. This section explores how GM modulation can enhance drug efficacy and reduce toxicity, as well as the potential for drug repurposing and combination therapies.

#### 5.4.1 GM-mediated efficacy and toxicity

The GM can modify the chemical structure of pharmaceutical compounds, influencing their pharmacodynamics. For example, certain gut bacteria can metabolize drugs, affecting their efficacy and toxicity (Klünemann et al., 2021). Modulating the GM to enhance drug efficacy and reduce toxicity is a promising strategy for improving TB treatment outcomes (Liu et al., 2025).

#### 5.4.2 Drug repurposing

Drug repurposing involves identifying new therapeutic uses for existing drugs. AI and machine learning algorithms can be used to identify potential drug targets and repurpose existing drugs for TB treatment. For example, comparative in silico analyses of host–pathogen metabolic pathways have identified potential MTB drug targets. By leveraging GM modulation, it may be possible to enhance the efficacy of repurposed drugs and reduce their toxicity.

#### 5.4.3 Combination therapies

Combination therapies involving multiple drugs can improve treatment outcomes by targeting different pathways and reducing the risk of drug resistance. GM modulation can be integrated with combination therapies to enhance their efficacy and reduce toxicity. For example, probiotics and prebiotics can be used alongside existing TB drugs to improve treatment outcomes and reduce adverse effects (Liu et al., 2025).

GM-targeted interventions offer great potential to optimize TB prognosis. Modulating gut microbiota via FMT, probiotics, prebiotics and dietary regulation can boost host immune function, alleviate inflammatory responses, and strengthen the effectiveness of conventional anti-TB treatments. Additionally, regulating key metabolic pathways, elevating vaccine immunogenicity, and combining microbiota modulation with pharmacological therapy can further improve the host’s defense against MTB infection. Further studies are warranted to optimize such intervention approaches and validate their safety and clinical efficacy.

## 6 Clinical trials of FMT and probiotics, challenges in translation, and regulatory considerations in the gut microbiota-tuberculosis interaction field

As a developing interdisciplinary area, the study of interactions between TB and the GM is at a pivotal point, moving from initial concept validation toward clinical application (Ma et al., 2022; Liu et al., 2025). Microbiome-based interventions, such as FMT and probiotics, are being explored as potential supplementary treatments alongside standard anti-tuberculosis therapy (ATT). Current research mainly aims to confirm the effectiveness of probiotics as adjunct therapies to reduce the side effects of ATT, followed by investigations into FMT for addressing severe gut dysbiosis. Most existing studies on FMT are small-scale, single-center, prospective exploratory trials and lack robust evidence from large, multicenter RCTs (Chai et al., 2025; Liu et al., 2025). This limitation may stem from the combined challenges of chronic infection and prolonged chemotherapy in TB patients, which lead to pathogen-specific patterns of gut microbiota disruption. Moreover, the direct suppressive effects of anti-TB medications—particularly rifampicin and isoniazid—on gut microbiota differ from those caused by other drug types (Baral et al., 2025). The immune restoration processes involved in TB, such as granuloma formation and shifts in Th1/Th2 balance, may present more complex immune environments compared to other diseases. A summary of previous intervention outcomes involving probiotics, FMT, dietary or nutritional supplements, and other microbiota-targeted approaches in TB patients or MTB-infected animal models is provided in **Table 8**.

**Table 8:** Results of existing microbiome-based interventions in tuberculosis research.

Intervene type	Research level	Study target	Intervention protocol	Primary outcome	Bacterial community-related findings	References
Probiotics	RCT	First-line treatment for pulmonary tuberculosis patients	Probiotics + B-complex Vitamins vs Standard Treatment	IFN-γ exhibited an upward trend in January and declined thereafter in February; IL-12 levels in both groups initially increased followed by a decrease, with a more pronounced rise observed in the intervention group.	No microbial community detected	(Suprapti et al., 2018)
Probiotics (subgroup analysis)	Forward-looking cohort (subgroup)	First-line treatment for pulmonary tuberculosis patients	VSL3 or Evogut combined with anti-tuberculosis therapy (within 2 months, based on physician prescription)	The alpha-diversity of the probiotic group showed significant improvement; β-diversity exhibited no notable changes.	Microbiome sequencing: Akkermansia was the dominant genus in untreated PTB patients; after probiotic therapy, Agathobacter, Fusobacterium, and Faecalibacterium were enriched.	(Baral et al., 2025)
FMT	Preclinical	BALB/c mice (Mtb H37Rv aerosol infection)	Wild mouse microbiota colonization vs. conventional laboratory mice	The bacterial load in the lungs of WildR mice was significantly lower than that in LabC mice; pulmonary pathological damage was also reduced.	The microbial diversity of WildR was significantly higher than that of LabC; MTB infection led to a rapid loss of diversity in LabC, whereas WildR maintained stable diversity.	(Xie et al., 2023)
FMT (logical inference)	Summarize	—	It is proposed that FMT may enhance anti-tuberculosis immunity by restoring gut microbiota homeostasis.	—	—	(Kang et al., 2026)
Dietary intervention (nutritional supplementation)	Clinical trial	Malnourished multidrug-resistant tuberculosis patients	High-energy, high-protein oral nutritional supplement for 2 months	Primary endpoint: Weight change; Secondary endpoints: Albumin, CRP, ESR	No microbial community detection was designed	(Isbaniah, 2025)
Dietary intervention (qualitative study)	Qualitative investigation	Family contacts of tuberculosis patients (India)	Nutritional supplementation (TB LION study)	Identification of factors that promote and hinder intervention adherence	No microbial community detected	(Carwile et al., 2025)
Complex intervention (microbiota regulation)	Summarize	—	Propose a potential application framework for “microbiome-directed therapies” such as probiotics, prebiotics, synbiotics, SCFAs, and FMT in tuberculosis.	—	—	(Kang et al., 2026)

RCT, Randomized controlled trial; FMT, Fecal microbiota transplantation.

### 6.1 Preclinical research on FMT, probiotics, and dietary approaches

Research on FMT in TB is currently confined to animal studies. One investigation demonstrated that colonizing experimental BALB/c mice with the diverse microbiota from wild mice led to significantly reduced bacterial loads in the lungs and less severe lung damage after aerosol exposure to MTB (Xie et al., 2023). This provides crucial experimental evidence supporting a causal link between the microbiota and TB. The study also showed that conventional laboratory mice experienced a rapid decline in microbial diversity following infection, whereas the microbiota of wild-derived mice remained stable. This offers direct proof that microbiota diversity underpins protective immunity against TB. Antibiotic-induced disruption of the microbiota was found to impair the intestinal barrier and cause structural damage to the lungs, with Clostridioides difficile infection further worsening lung pathology. In the context of C. difficile infection, human macrophages lost their ability to engulf MTB, and expression of SLAMF1 was reduced. This represents the first cellular-level evidence of a direct interference by an intestinal pathogen on pulmonary anti-TB immune responses. Additionally, Chinese researchers have shown that Bacteroides fragilis strain BF839 alleviates liver injury caused by anti-TB drugs in mice by reducing inflammation and modulating the gut microbiota (Li et al., 2025). Depletion of the microbiota due to antibiotics increased mice’s susceptibility to MTB infection, whereas FMT lowered bacterial loads (Xie et al., 2023).

### 6.2 Clinical evidence for FMT and probiotics

Research on the relationship between probiotics and TB shows that probiotic supplementation can help reduce gastrointestinal side effects caused by anti-TB medications in patients and may also help counteract antibiotic-induced disturbances in gut microbiota (Baral et al., 2026). However, this particular study did not examine fecal microbiota, so it did not clarify the microbiota-related mechanisms involved. Another study that combined probiotic supplementation with B vitamins during intensive treatment observed that IFN-γ levels tended to rise after one month but significantly dropped by the second month, possibly indicating a shift from early Th1-driven immune benefits to more complex immune system recovery in later treatment phases (Lin et al., 2020). Patients with PTB who received probiotics (n=5) showed a significant increase in gut microbiota alpha diversity compared to those who did not receive probiotics, although there was no notable change in beta diversity (Baral et al., 2022). This suggests that short-term probiotic use may guide the microbiota in a specific direction—such as increasing butyrate-producing bacteria like Agathobacter and Faecalibacterium—but is insufficient to fully reverse the broad microbial community changes associated with PTB. Unlike the relatively active research on probiotics, clinical studies on FMT in TB are lacking, likely due to the complexity of the procedure and the higher risk of infection involved in transplanting donor fecal material into a recipient’s gut.

### 6.3 Challenges and clinical translational barriers for FMT, probiotics, and dietary interventions

There are several scientific and clinical challenges hindering the widespread clinical application of microbiome-based therapies for TB. Although a link between gut dysbiosis and TB has been established, the precise molecular mechanisms remain unclear (Nasare and Bagade, 2025). The potential benefits of probiotics, FMT, and dietary fibers (such as short-chain fatty acids) in TB are often inferred from studies in other conditions like inflammatory bowel disease (IBD), cancer immunotherapy, and more. However, this extrapolation is based on limited evidence because IBD is an autoimmune disease primarily driven by Th17 immune responses, which differ significantly from the Th1/Th2 balance and granuloma-based immunity seen in TB. Similarly, cancer patients typically experience immunosuppression or immune tolerance, which contrasts with the moderately activated immune response needed to fight active TB. Probiotic research has notable limitations: most studies have not analyzed fecal microbiota or immune markers, so even when clinical improvements are observed, it is unclear whether these benefits result from enhanced host metabolism or from immune modulation via changes in gut microbiota or their metabolites. Therefore, a clear causal pathway linking microbiota changes to immune modulation and clinical outcomes has yet to be established. While preclinical data on FMT are promising, applying FMT in TB patients poses unique difficulties. There is currently no standardized clinical trial design for FMT in TB, and existing studies vary widely in probiotic strains used, dosages, timing of intervention, and outcome measures (Baral et al., 2026). Moreover, high-quality, large-scale, multicenter trials confirming FMT’s effectiveness are lacking. TB patients undergoing long-term multidrug chemotherapy experience continuous “drug pressure” on their gut microbiota, which may create strong resistance to colonization by transplanted microbes. Additionally, the safety of FMT must be taken into account. Patients with active TB often have weakened immune systems, and live biotherapeutic products carry risks such as bacterial translocation and infections like bacteremia. This necessitates caution from regulatory authorities and healthcare providers when developing live biotherapeutic treatments.

Microbiome-based therapies, including FMT and probiotics, require stringent and effective regulatory frameworks. This includes implementing thorough ethical reviews and standardized operational procedures. Before human trials can begin, comprehensive preclinical evaluations of safety and efficacy must be completed. Collaboration among multidisciplinary organizations and regulatory agencies is essential to develop consensus-driven standardized protocols. These should cover donor screening for FMT, preparation and storage guidelines, viability and stability criteria for probiotic products, and dosing schedules when used alongside anti-TB drugs. A comprehensive safety and monitoring system integrating microbiota, metabolites, immune responses, and clinical outcomes should be established. The main challenge in microbiome intervention research for TB lies in the gap between rapid advances in understanding the gut–lung axis mechanisms and the slow progress in clinical intervention evidence. Future research should prioritize two key areas: First, routinely include microbiota analysis as an outcome measure in clinical trials. Whether testing probiotics or dietary changes, studies should incorporate fecal microbiota profiling (using 16S rRNA sequencing or metagenomics) and metabolite assessments (such as short-chain fatty acids) to build a complete evidence chain linking interventions to microbiota changes, immune responses, and clinical results. Second, launch TB-specific clinical trials of FMT. Based on strong preclinical findings, small-scale, single-arm, dose-escalation feasibility studies should be conducted to assess the safety and effectiveness of FMT in patients with multidrug-resistant TB or those suffering severe side effects from current treatments.

## 7 Controversies and open scientific problems in GM-TB research

### 7.1 Controversial issues in research on the GM and TB

Many fundamental and clinical studies have demonstrated that the development and progression of TB are frequently linked with imbalances in the GM. However, considerable variability in baseline characteristics of study cohorts, differences in microbial detection methods, and geographic diversity among study populations have resulted in significant inconsistencies in findings across research groups, greatly impeding the clinical application of these results. A major point of debate concerns the specific changes in gut microbial diversity observed in TB patients. Most research suggests that individuals with ATB show a marked reduction in both alpha and beta diversity of gut microbes, with diversity levels inversely related to disease severity and rates of treatment failure. Nonetheless, these changes do not follow a consistent pattern but instead exhibit strong geographic variation, with differing trends in microbial diversity reported in cohort studies from various regions (Mbabazi et al., 2025). Some studies have reported that TB onset is closely linked to reduced diversity in lung microbiota, while gut microbial diversity remains largely unchanged. In contrast, anti-TB therapy can cause a sustained decrease in microbial diversity in both the lungs and gut, leading to depletion of key dominant bacterial genera in the gut and disruption of gut ecological balance (Mbabazi et al., 2025). The prolonged effects of anti-TB treatment on the GM are also debated. Traditionally, it is believed that the broad-spectrum antimicrobial properties of anti-TB drugs induce temporary GM disturbances, which delay immune function recovery, but that the GM structure fully recovers within six months after treatment completion. However, recent longitudinal studies have found that in some patients, the abundance and composition of various gut bacterial groups remain significantly different from those in healthy controls even 15 months post-treatment, indicating that the impact of anti-TB therapy on the GM may be long-lasting (Diallo et al., 2023). In summary, preliminary evidence suggests that anti-TB treatment has a significant and enduring regulatory effect on the GM. Yet, the reversibility of these long-term effects, their precise magnitude, and associated clinical outcomes—such as influence on relapse risk and long-term immune function—require further investigation with larger sample sizes and extended follow-up periods.

### 7.2 Differentiating correlation from causation in gut microbiome and tuberculosis studies

At present, most research in this area remains descriptive, establishing only associations between the GM and TB without confirming causal relationships through rigorous experimental approaches. Studies have shown that patients with active TB exhibit characteristic GM imbalances, mainly characterized by a reduction in beneficial commensal bacteria (such as Bifidobacterium and Lactobacillus) and an increase in bacteria linked to inflammation (including certain genera within Proteobacteria). The severity of GM disruption correlates significantly with sputum smear conversion time, the occurrence of adverse reactions to anti-TB drugs, and systemic inflammatory cytokine levels (e.g., TNF-α, IL-6). Moreover, anti-TB treatment dynamically alters the composition and structure of the GM, with changes in gut microbial communities occurring in parallel with clinical symptom improvement and immune function recovery, further suggesting a close association.

Current research exploring the relationship between the GM and TB still faces three fundamental unresolved scientific questions: (1) Does GM imbalance predispose individuals to TB, or is it a secondary effect resulting from metabolic and immune disturbances after infection with MTB? (2) Do specific gut microbes directly influence the onset and progression of MTB infection, or do they simply act as biological markers of immune dysfunction without actively regulating TB infection? (3) Can interventions aimed at restoring GM balance effectively improve clinical outcomes for TB patients, such as shortening treatment duration, lowering relapse rates, or reducing adverse treatment effects? There is also debate within the scientific community regarding causality. It is generally accepted that there is a two-way interaction between the GM and TB: gut dysbiosis can increase susceptibility to MTB by affecting innate and adaptive immunity, while MTB infection and anti-TB treatments can further damage the gut barrier and disrupt microbiome balance, creating a complex feedback loop (Liu et al., 2025). However, genetic studies using Mendelian Randomization (MR) suggest that, at the molecular genetic level, the GM’s influence on TB risk may be more fundamental than the reverse, providing indirect evidence that the GM may play a causal role in TB development (Yuan et al., 2024).

Currently, research into the causal mechanisms linking the GM and TB has three main limitations: first, there is a lack of large-scale, multi-center randomized controlled trials (RCTs) in humans to robustly assess the impact of GM interventions (such as probiotics or FMT) on TB clinical outcomes; second, the bidirectional regulatory mechanisms of the gut-lung axis are not fully understood, especially the molecular processes by which MTB infection and systemic inflammation disrupt the gut mucosal barrier and microbiome balance; third, the detailed molecular pathways of the “gut microbiota-metabolite-immunity loop-MTB regulation” remain unclear, leaving the specific ways in which gut microbial metabolites affect MTB survival and replication through host immune modulation unresolved.

### 7.3 Gaps in gut microbiome and tuberculosis research

There are significant gaps in current research on the GM and TB, mainly across four areas: mechanistic understanding, translational applications, clinical studies, and technical standardization.

At the mechanistic level, key gaps include: unclear cellular and molecular pathways by which the gut-lung axis mediates interactions between the GM and pulmonary MTB infection, particularly how gut microbes and their metabolites travel along this axis to influence lung immune responses; undefined strain-level functional specificity of gut microbes, with no effective identification yet of strains that directly inhibit MTB growth or replication; unclear regulatory roles and mechanisms of the GM in tuberculous granuloma formation, latent TB reactivation, and host genetic susceptibility; and a lack of systematic characterization of the immune-metabolic crosstalk between the GM and TB-related immune responses, necessitating further investigation into their interaction targets and regulatory patterns.

At the level of translational applications, significant gaps are apparent: there is a lack of rigorously validated, non-invasive, and highly specific GM biomarkers capable of accurately distinguishing active TB, latent TB infection, and non-tuberculous mycobacterial (NTM) infections. Additionally, no standardized protocol exists for GM interventions in TB patients—particularly those with drug-resistant TB—resulting in inconsistent dosing, timing, and effectiveness criteria for probiotics, prebiotics, and FMT. The influence of the GM on the metabolism, efficacy, and toxicity of anti-TB drugs remains largely unexplored, necessitating further investigation into their interaction mechanisms. Research into using GM modulation to boost the immune response to BCG and next-generation TB vaccines is still in its early stages, with feasibility and specific regulatory protocols requiring more study.

At the clinical research level, current shortcomings include the predominance of cross-sectional studies, with a lack of large-scale, long-term longitudinal follow-ups and multi-center randomized controlled trials (RCTs), making it difficult to track dynamic changes in the GM throughout the entire course of TB. There is also a severe shortage of GM data from special populations such as children, pregnant women, and patients co-infected with HIV-TB or diabetes-TB, which limits understanding of interactions in these groups. Moreover, studies rarely cover the full disease progression—from latent TB infection through active disease onset, treatment, cure, and relapse—hindering the ability to uncover intrinsic links between GM alterations and TB progression.

At the technical and standardization level, key gaps include the absence of unified standard operating procedures for GM sample collection, preservation, sequencing, and bioinformatics analysis. This lack of standardization reduces comparability across studies and impedes comprehensive meta-analyses. There is also a shortage of affordable, rapid, point-of-care GM detection technologies suitable for resource-limited settings, restricting research in primary care environments. Furthermore, artificial intelligence-based GM prediction models (such as those for TB diagnosis and prognosis) suffer from limited interpretability and lack prospective clinical validation, which hinders their clinical application.

In summary, research on the GM and TB has advanced from initial descriptive correlation studies to early causal inference stages but still faces major challenges, including conflicting results, unclear causality, and barriers to clinical translation. While animal models and Mendelian randomization studies have preliminarily demonstrated a causal regulatory role of the GM in TB susceptibility, evidence from human intervention studies remains limited. Future research should prioritize establishing standardized multi-center cohorts to reduce heterogeneity; deeply exploring the core regulatory mechanisms of the gut-lung axis and decoding the “gut microbiota-metabolite-immunity” network; conducting RCTs targeting the GM to confirm the clinical effectiveness of intervention strategies; and systematically analyzing the biological functions of gut microbes at the strain level. Addressing these gaps will help position the GM as a clinically actionable target for precise global TB prevention, early diagnosis, and personalized treatment optimization, offering new strategies and insights for TB control.

## 8 Conclusions and challenges

**Figure 5:**
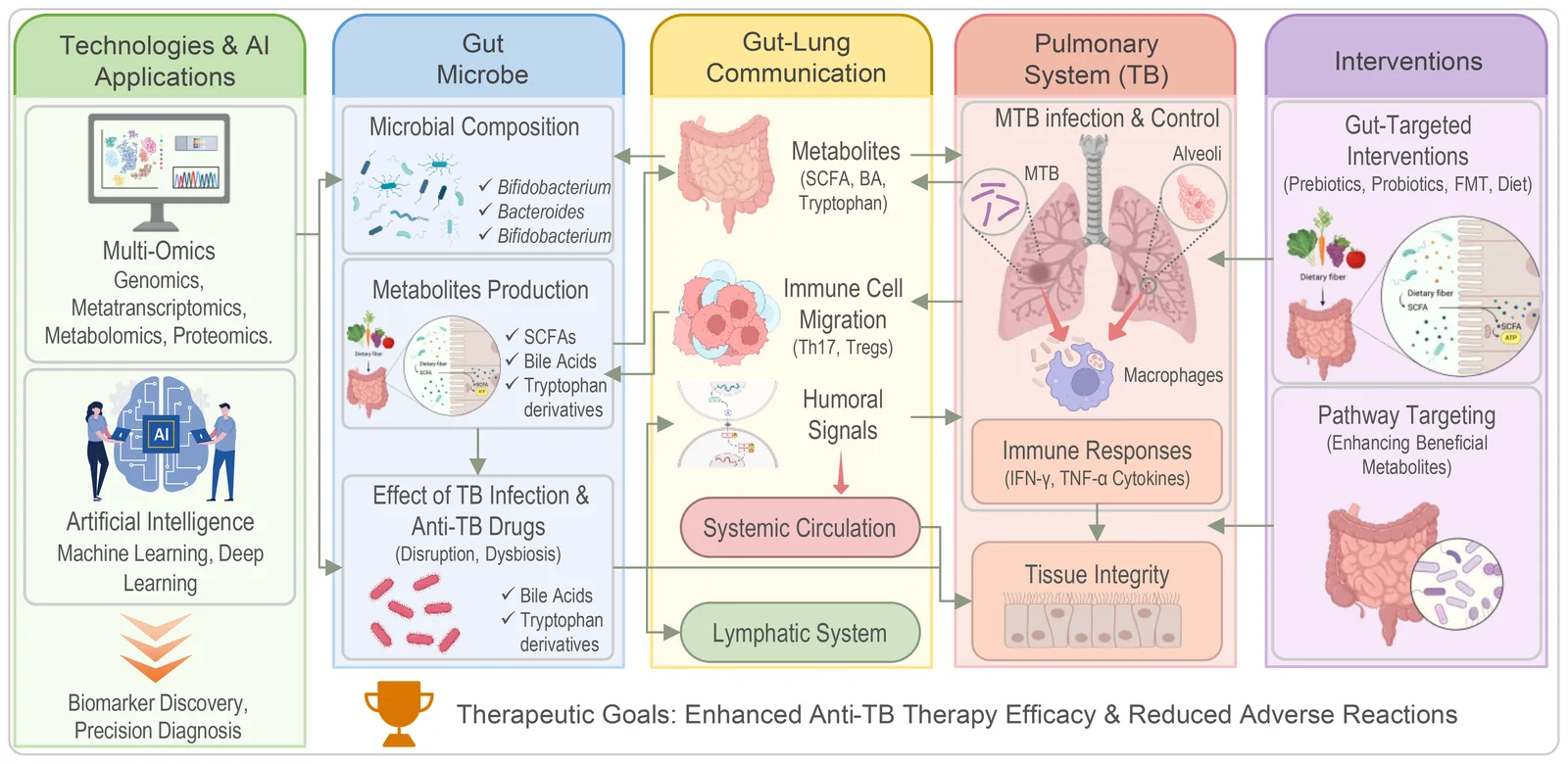
Integrated schematic of the gut-lung axis in TB. This comprehensive framework illustrates five interconnected components: technologies and AI applications, gut microbiome, gut-lung communication, pulmonary system and interventions. The overarching therapeutic goals are to enhance anti-TB therapy efficacy and reduce adverse reactions.

The integration of cutting-edge technologies such as NGS, metabolomics, proteomics, and AI have significantly advanced our understanding of the complex interplay between the GM and TB. These advancements hold the promise of transforming TB diagnosis, treatment, and prevention. However, several challenges remain in translating these technological breakthroughs into effective clinical strategies. This section addresses the pathways for technological integration, ethical considerations, and future research priorities in the field of GM-TB research (**Figure 5**).

### 8.1 Pathways for technological integration: standardization, cross-center validation, and data sharing

The successful translation of GM-TB research into clinical practice requires robust technological integration and validation. Standardization of methodologies across different research centers is crucial for generating reproducible and comparable data (Zhang et al., 2023). This includes the use of standardized protocols for sample collection, processing, and analysis, as well as the adoption of common bioinformatics pipelines for data interpretation. Cross-center validation studies are essential for confirming the generalizability of findings and ensuring that interventions are effective across diverse populations. Additionally, data sharing platforms facilitate the aggregation of large datasets, enabling researchers to conduct comprehensive analyses and identify robust biomarkers and therapeutic targets. The establishment of consortia and collaborative networks can further enhance the efficiency of data sharing and validation efforts.

### 8.2 Ethics and accessibility: privacy protection, low-cost detection, and resource inequality

The deployment of advanced technologies in TB care raises several ethical considerations, particularly regarding data privacy and accessibility. Ensuring the protection of patient data is paramount, especially in the context of multi-omics studies that involve sensitive clinical and personal information (Seyed Aria and Alexander, 2024).

Robust data governance frameworks must be in place to safeguard participant privacy while promoting open data sharing for scientific advancement. Additionally, the development of low-cost, high-performance diagnostic tools is essential for ensuring that these technologies are accessible in low-resource settings, where the burden of TB is highest. Addressing resource inequalities and ensuring equitable access to advanced diagnostics and treatments are critical challenges that must be addressed through global collaboration and investment in healthcare infrastructure.

### 8.3 Future research priorities: causal inference, real-time monitoring, and global multicenter clinical trials

Future research in GM-TB should focus on establishing causal relationships between the GM and TB outcomes, moving beyond correlative associations. Causal inference techniques can help identify true causal pathways and inform the development of targeted interventions. Real-time monitoring technologies, such as wearable devices and point-of-care diagnostics, offer the potential for dynamic tracking of disease progression and treatment response, enabling timely interventions and personalized care (Mahomed et al., 2019). Finally, global multicenter clinical trials are essential for validating the efficacy and safety of GM-targeted interventions across diverse populations and settings. These trials should be designed to address the specific needs of high-burden regions and ensure that the benefits of GM-TB research are realized globally.

In summary, the integration of advanced technologies in GM-TB research has opened new avenues for understanding and combating TB. However, the successful translation of these technologies into clinical practice requires addressing significant challenges related to standardization, data privacy, and accessibility. Future research must prioritize causal inference, real-time monitoring, and global collaboration to ensure that the benefits of GM-TB research are realized worldwide. By addressing these challenges and leveraging the potential of technological advancements, we can move closer to effective strategies for the prevention, diagnosis, and treatment of TB.
