# Correction: Harnessing the gut microbiome to combat tuberculosis: a technological and clinical review

**DOI:** 10.3389/fcimb.2026.1961302

**Published:** 2026-10-02

**Authors:** Weiguo Sun, Meng Xiao, Syed Luqman Ali, Chanyuan Jin, Asifullah Khan, Shakirullah, Ruizi Ni, Yajing An, Mingming Zhang, Yuan Tian, Shradha Kaushik, Yuhang Zhang, Wenping Gong

**Affiliations:** 1 Beijing Key Laboratory of Emergency Vaccine Development and Process Translation, Senior Department of Tuberculosis, Chinese PLA General Hospital, Beijing, China; 2 Computer Network Information Center, Chinese Academy of Sciences, Beijing, China; 3 DUKE-NUS Medical School, National University of Singapore, Singapore, Singapore; 4 Department of Biochemistry, Abdul Wali Khan University Mardan, Mardan, KPK, Pakistan; 5 2nd Dental Center, Peking University School and Hospital of Stomatology, Beijing, China; 6 Department of Zoology, Abdul Wali Khan University Mardan, Mardan, KPK, Pakistan; 7 Graduate School, Hebei North University, Zhangjiakou, Hebei, China; 8 Department of Biomedical Sciences, University of Windsor, Windsor, ON, Canada; 9 Institute of Clinical Pharmacology, Peking University First Hospital, Beijing, China

**Keywords:** artificial intelligence (AI), gut microbiome (GM), metabolomics, next-generation sequencing (NGS), proteomics, tuberculosis (TB)

In the published article, the reference for [Haitjema et al.,2026] was erroneously written as [Haitjema, A., Zhang, J., van Beijnum, B. F., Veltink, P. H., Reenalda, J., and Wouda, F. J. (2026). Assessing the usability of the angular momentum method for estimating wholebody inertia in humans: a case study. Med. Eng. Phys. 147 (4). doi: 10.1088/1873-4030/ae52fa]. It should be [Suprapti, B., Suharjono, Raising, R., Yulistiani, Izzah, Z., Nilamsari, W. P., Wulaningrum, P. A., & Bachtiar, A. (2018). Effects of probiotics and Vitamin B supplementation on IFN-γ and IL-12 levels during intensive phase treatment of tuberculosis. Indonesian Journal of Pharmacy 29 (2), 80–85. doi: 10.14499/indonesianjpharm29iss2pp80].

The reference for [Ali et al., 2025] was erroneously written as [Ali, A., Ali, S. L., Alamri, A., Khatrawi, E. M., Baidussenova, A., Suleimenova, F., Mishra, V. K., Khan, A., Dusmagambetov, M., Askarova, G. Multi-epitope-based vaccine models prioritization against Astrovirus MLB1 using immunoinformatics and reverse vaccinology approaches. Journal, genetic engineering & biotechnology 2025, *23*, 100451. doi: 10.1016/j.jgeb.2024.100451]. It should be [Simwela, N. V., Jaecklein, E., Sassetti, C. M., Russell, D. G. (2025). Impaired fatty acid import or catabolism in macrophages restricts intracellular growth of Mycobacterium tuberculosis. Elife 13, RP102980. doi: 10.7554/eLife.102980].

The reference for [Ali et al., 2023] was erroneously written as [Ali, S. L., Ali, A., Alamri, A., Baidussenova, A., Dusmagambetov, M., Abduldayeva, A. Genomic annotation for vaccine target identification and immunoinformatics-guided multi-epitope-based vaccine design against Songling virus through screening its whole genome encoded proteins. Frontiers in immunology 2023, *14*, 1284366. doi: 10.3389/fimmu.2023.1284366]. It should be [Liu X., Yang M. J., Xu P., Du M. W., Li S. S., Jin Shi J., et al. (2024). Kynurenine-AhR reduces T-cell infiltration and induces a delayed T-cell immune response by suppressing the STAT1-CXCL9/CXCL10 axis in tuberculosis. Cell Mol. Immunol. 21 (12), 1426–1440. doi: 10.1038/s41423-024-01230-1].

The reference for [Martínez and Ruiz, 2019] was erroneously written as [Martínez, C. R., Ruiz, C. J. Alterations in Host Lipid Metabolism Produced During Visceral Leishmaniasis Infections. Current tropical medicine reports 2019, *6*, 250–255. doi: 10.1007/s40475-019-00187-w]. It should be [Lee W., Ven B. C. V., Fahey R. J., Russell D. G. (2013). Intracellular Mycobacterium tuberculosis Exploits Host-derived Fatty Acids to Limit Metabolic Stress. J. Biol. Chem. 8;288 (10), 6788–6800. doi: 10.1074/jbc.M112.445056].

The reference for [Ke et al., 2023] was erroneously written as [Ke, B., Gong, R., Shen, A., Qiu, H., Chen, H., Zhang, Z., Li, W., Xie, Y., Li, H. Risk stratification algorithm for clinical outcomes in anemic patients undergoing percutaneous coronary intervention. Annals of medicine 2023, 55, 2249200. doi: 10.1080/07853890.2023.2249200]. It should be [Nyangahu D. D., Happel A. U., Wendoh J., Kiravu A., Wang, Y., Feng, C., et al. (2023). Bifidobacterium infantis associates with T cell immunity in human infants and is sufficient to enhance antigen-specific T cells in mice. Sci. Adv. 8;9 (49), eade1370. doi: 10.1126/sciadv.ade1370].

The reference for [Rasoamanana et al., 2023] was erroneously written as [Rasoamanana, H., Ravelomanantsoa, S., Nomenjanahary, M. V., Gauche, M. M., Prior, P., Guérin, F., Robène, I., Pecrix, Y., Poussier, S. Bacteriocin Production Correlates with Epidemiological Prevalence of Phylotype I Sequevar 18 Ralstonia pseudosolanacearum in Madagascar. Applied and environmental microbiology 2023, *89*, e0163222. doi: 10.1128/aem.01632-22]. It should be [Jeyanathan M., Vaseghi-Shanjani M., Afkhami S., Grondin, J. A., Kang, A., D’Agostino, M. R., et al. (2022). Parenteral BCG vaccine induces lung-resident memory macrophages and trained immunity via the gut-lung axis. Nat. Immunol. 23 (12), 1687–1702. doi: 10.1038/s41590-022-01354-4].

The reference for [Liu et al., 2024] was erroneously written as [Liu, B., Xing, Z., Xue, Y., Zhang, J., Zhai, J. Effect of Pyrolysis Temperature on the Carbon Sequestration Capacity of Spent Mushroom Substrate Biochar in the Presence of Mineral Iron. Molecules 2024, *29*. doi: 10.3390/molecules29235712]. It should be [Kang K., Kim J. Y., Yim J. J., Kim D. (2026). Gut-lung axis and microbiome alterations in mycobacterial infections: from pathogenesis to therapeutic potential. Gut Microbes 31;18 (1), 2612428. doi: 10.1080/19490976.2025.2612428].

The reference for [Zhong et al., 2024] was erroneously written as [Zhong, H., Song, Y., Long, F., Lu, H., Ai, M., Li, T., Yao, Y., Sakai, Y., Ikeda, M., Takahashi, K., et al. Design of Excellent Mechanical Performances and Magnetic Refrigeration via *In Situ* Forming Dual-Phase Alloys. Advanced materials 2024, *36*, e2402046. doi: 10.1002/adma.202402046]. It should be [Isbaniah, F. Oral Nutritional Supplementation and Weight Changes During Therapy in Malnourished Patients With Resistant Tuberculosis: A Clinical Trial. Available online at: https://ichgcp.net/clinical-trials-registry/NCT07058233?hl=zh# (Accessed May 17, 2026)].

The reference for [Mitsui et al., 2022] was erroneously written as [Mitsui, T., Kadota, T., Wakabayashi, M., Nakajo, K., Shinmura, K., Sunakawa, H., Sato, D., Minamide, T., Takashima, K., Murano, T., et al. Factors of technical difficulty in conventional and traction-assisted esophageal endoscopic submucosal dissection. Esophagus: official journal of the Japan Esophageal Society 2022, *19*, 452–459. doi: 10.1007/s10388-022-00913-w]. It should be [Baral T., Fayaz S. M. A., Manu M. K., Kudru, C. U., Singh, J., Mukhopadhyay, C., et al. (2025). Gut microbiota profile in newly diagnosed pulmonary tuberculosis patients: an exploratory pilot study in southern India. Gut Pathog. 11;17 (1), 59. doi: 10.1186/s13099-025-00736-x].

The reference for [Yan et al., 2022] was erroneously written as [Yan, C., Wang, L., Lin, J., Xu, J., Zhang, T., Qi, J., et al. (2022). A fully automatic artificial intelligence–based CT image analysis system for accurate detection, diagnosis, and quantitative severity evaluation of pulmonary tuberculosis. Eur. Radio 32, 2188–2199. doi: 10.1007/s00330-021-08365-z]. It should be [Gong, W., Liang, Y., Wu, X. (2018). The current status, challenges, and future developments of new tuberculosis vaccines. Hum. Vacc. Immunother. 14, 1697–1716. doi: 10.1080/21645515.2018.1458806.].

The reference for [Sharma et al., 2021] was erroneously written as [Sharma, A., Sharma, A., Malhotra, R., Singh, P., Chakrabortty, R. K., Mahajan, S., et al. (2021). An accurate artificial intelligence system for the detection of pulmonary and extra pulmonary tuberculosis. Tuberculosis 131, 102143. doi: 10.1016/j.tube.2021.102143]. It should be [Gong, W., Pan, C., Cheng, P., Wang, J., Zhao, G., Wu, X. (2022). Peptide-Based Vaccines for Tuberculosis. Front. Immunol. 13, 830497. doi: 10.3389/fimmu.2022.830497.].

The reference for [Du et al., 2024] was erroneously written as [Du, J., Su, Y., Qiao, J., Gao, S., Dong, E., Wang, R., et al. (2024). Application of artificial intelligence in diagnosis of pulmonary tuberculosis. Chin. Med. J. 137, 559–561. doi:10.1097/CM9.0000000000003018]. It should be [An, Y., Ni, R., Zhuang, L., Yang, L., Ye, Z., Li, L., et al. (2025). Tuberculosis vaccines and therapeutic drug: challenges and future directions. Mol. Biomed. 6, 4. doi: 10.1186/s43556-024-00243-6.].

The reference for [Spiegel et al., 2021] was erroneously written as [Spiegel, J. M., Ehrlich, R., Yassi, A., Riera, F., Wilkinson, J., Lockhart, K., et al. (2021). Using artificial intelligence for high-volume identification of silicosis and tuberculosis: a bio-ethics approach. Ann. Global Health 87, 58. doi: 10.5334/aogh.3206]. It should be [Young C, Barker S, Ehrlich R, Kistnasamy B, Yassi A. (2020). Computer-aided detection for tuberculosis and silicosis in chest radiographs of gold miners of South Africa. Int. J. Tuberc. Lung. Dis. 24 (4), 444–451. doi: 10.5588/ijtld.19.0624].

The reference for [Mai and Draganov, 2009] was erroneously written as [Mai, V., and Draganov, P. V. (2009). Recent advances and remaining gaps in our knowledge of associations between gut microbiota and human health. World J. Gastroenterol. 15, 81–85. doi: 10.3748/wjg.15.81]. It should be [Woodworth, M. H., Conrad, R. E., Haldopoulos, M., Pouch, S. M., Babiker, A., Mehta, A. K., et al. (2023). Fecal microbiota transplantation promotes reduction of antimicrobial resistance by strain replacement. Sci. Transl. Med. 15, eabo2750. doi: 10.1126/scitranslmed.abo2750.].

In addition, there was an error. [In page 13, “Wu et al. utilized 3D convolutional neural networks (3D-CNNs) …”

A correction has been made to [4 AI-driven biomarker discovery and diagnostic decision-making], *[4.2 AI in TB diagnosis and treatment prediction*], [Page 13]. This sentence previously stated:

“[Wu et al. utilized 3D convolutional neural networks (3D-CNNs) …]”

The corrected sentence appears below:

“[Li et al. utilized 3D convolutional neural networks (3D-CNNs) …]”

Also, there was an error. [In page 13, “Jerry et al. developed an ML based computer-aided diagnosis (CAD) model to distinguish between silicosis and TB in South African gold miners; the model was evaluated in real-world clinical settings and achieved a sensitivity of 90.1% and a specificity of 80.3%, demonstrating its practical value in improving diagnostic efficiency (Spiegel et al., 2021).”

A correction has been made to [4 AI-driven biomarker discovery and diagnostic decision-making], *[4.2 AI in TB diagnosis and treatment prediction*], [Page 13]. This sentence previously stated:

“[Jerry et al. developed an ML based computer-aided diagnosis (CAD) model to distinguish between silicosis and TB in South African gold miners; the model was evaluated in real-world clinical settings and achieved a sensitivity of 90.1% and a specificity of 80.3%, demonstrating its practical value in improving diagnostic efficiency (Spiegel et al., 2021).]”

The corrected sentence appears below:

“[Young et al. developed an ML based computer-aided diagnosis (CAD) model to distinguish between silicosis and TB in South African gold miners; the model was evaluated in real-world clinical settings and achieved a sensitivity and specificity of 98.2%, demonstrating its practical value in improving diagnostic efficiency (Young et al., 2020)]”

There was also an error. [In page 16, the “Table 7” of the sentence “Focusing on specific metabolic pathways allows for precise adjustment of the gut microbiota, which can lead to better outcomes in TB (Table 7).”

A correction has been made to [5 Intervention strategies based on the GM], *[5.2 Targeted metabolic pathways: SCFA precursors, bile acid receptor agonists, and IDO inhibitors*], [Page 16]. This sentence previously stated:

“[Focusing on specific metabolic pathways allows for precise adjustment of the gut microbiota, which can lead to better outcomes in TB (Table 7).]”

The corrected sentence appears below:

“[Focusing on specific metabolic pathways allows for precise adjustment of the gut microbiota, which can lead to better outcomes in TB.]”

There was an error in [**Table 8** Results of existing microbiome-based interventions in tuberculosis research] as published. [In **Table 8**, the “Research level” column contains two inaccuracies: For the entry labelled “Commentary” (fourth row), the correct term should be “Summarize”. For the entry labelled “Clinical test” (fifth row), the correct term should be “Clinical Trial”. Equally, the “Reference” column contains four inaccuracies: On the first row, the reference appears as (Haitjema et al., 2026), but the correct reference should be (Suprapti et al., 2018). On the fourth row, the reference appears as (Liu et al., 2024) but the correct reference should be (Kang et al., 2026). On the fifth row, the reference appears as (Zhong et al., 2024) but the correct reference should be (Isbaniah, 2025). And on the seventh row, the reference appears as (Liu et al.,2024) but the correct reference should be (Kang et al., 2026).].

The corrected [**Table 8]** appears below.

**Table 8:** Results of existing microbiome-based interventions in tuberculosis research.

Intervene type	Research Level	study target	Intervention Protocol	Primary Outcome	Bacterial community-related findings	References
Probiotics	RCT	First-line treatment for pulmonary tuberculosis patients	Probiotics + B-complex Vitamins vs Standard Treatment	IFN-γ exhibited an upward trend in January and declined thereafter in February; IL-12 levels in both groups initially increased followed by a decrease, with a more pronounced rise observed in the intervention group.	No microbial community detected	(Suprapti et al., 2018)
Probiotics (subgroup analysis)	Forward-looking cohort (subgroup)	First-line treatment for pulmonary tuberculosis patients	VSL3 or Evogut combined with anti-tuberculosis therapy (within 2 months, based on physician prescription)	The alpha-diversity of the probiotic group showed significant improvement; β-diversity exhibited no notable changes.	Microbiome sequencing: Akkermansia was the dominant genus in untreated PTB patients; after probiotic therapy, Agathobacter, Fusobacterium, and Faecalibacterium were enriched.	(Baral et al., 2025)
FMT	Preclinical	BALB/c mice (Mtb H37Rv aerosol infection)	Wild mouse microbiota colonization vs. conventional laboratory mice	The bacterial load in the lungs of WildR mice was significantly lower than that in LabC mice; pulmonary pathological damage was also reduced.	The microbial diversity of WildR was significantly higher than that of LabC; MTB infection led to a rapid loss of diversity in LabC, whereas WildR maintained stable diversity.	(Xie et al., 2023)
FMT (Logical Inference)	Summarize	—	It is proposed that FMT may enhance anti-tuberculosis immunity by restoring gut microbiota homeostasis.	—	—	(Kang et al., 2026)
Dietary Intervention (Nutritional Supplementation)	Clinical Trial	Malnourished multidrug-resistant tuberculosis patients	High-energy, high-protein oral nutritional supplement for 2 months	Primary endpoint: Weight change; Secondary endpoints: Albumin, CRP, ESR	No microbial community detection was designed	(Isbaniah, 2025)
Dietary Intervention (Qualitative Study)	Qualitative investigation	Family contacts of tuberculosis patients (India)	Nutritional supplementation (TB LION study)	Identification of factors that promote and hinder intervention adherence	No microbial community detected	(Carwile et al., 2025)
Complex Intervention (Microbiota Regulation)	Summarize	—	Propose a potential application framework for “microbiome-directed therapies” such as probiotics, prebiotics, synbiotics, SCFAs, and FMT in tuberculosis.	—	—	(Kang et al., 2026)

The original version of this article has been updated.

